# Single-Walled Carbon Nanotube (SWCNT) Loaded Porous Reticulated Vitreous Carbon (RVC) Electrodes Used in a Capacitive Deionization (CDI) Cell for Effective Desalination

**DOI:** 10.3390/nano8070527

**Published:** 2018-07-13

**Authors:** Ali Aldalbahi, Mostafizur Rahaman, Mohammed Almoiqli, Abdelrazig Hamedelniel, Abdulaziz Alrehaili

**Affiliations:** 1 Department of Chemistry, College of Science, King Saud University, Riyadh 11451, Saudi Arabia; mrahaman@ksu.edu.sa (M.R.); aelfaki@ksu.edu.sa (A.H.); 436107406@student.ksu.edu.sa (A.A.); 2 Nuclear Sciences Research Institute, King Abdulaziz City for Science and Technology, Riyadh 11442, Saudi Arabia; almoiqli@kacst.edu.sa

**Keywords:** RVC/a-SWCNT composite electrode, capacitive deionization, water purification, desalination technology

## Abstract

Acid-functionalized single-walled carbon nanotube (a-SWCNT)-coated reticulated vitreous carbon (RVC) composite electrodes have been prepared and the use of these electrodes in capacitive deionization (CDI) cells for water desalination has been the focus of this study. The performance of these electrodes was tested based on the applied voltage, flow rate, bias potential and a-SWCNT loadings, and then evaluated by electrosorption dynamics. The effect of the feed stream directly through the electrodes, between the electrodes, and the distance between the electrodes in the CDI system on the performance of the electrodes has been investigated. The interaction of ions with the electrodes was tested through Langmuir and Freundlich isotherm models. A new CDI cell was developed, which shows an increase of 23.96% in electrosorption capacity compared to the basic CDI cells. Moreover, a comparison of our results with the published results reveals that RVC/a-SWCNT electrodes produce 16 times more pure water compared to the ones produced using only CNT-based electrodes. Finally, it can be inferred that RVC/a-SWCNT composite electrodes in newly-developed CDI cells can be effectively used in desalination technology for water purification.

## 1. Introduction

Capacitive deionization (CDI) technology has become a new desalination technology developed in recent years with advantages such as energy saving, no secondary pollution, and simple operation, showing a broad prospect that this low-pressure and non-membrane desalination technology employs the basic electrochemical principle of adsorbing ions to high surface-area electrodes in a capacitive fashion such that the outgoing stream becomes devoid of the ions that were present in the incoming stream. The realization of high removal efficiency for capacitive deionization is strongly correlated with the high capacitance value, large surface area for ion accumulation, good electrical conductivity, and suitable pore size of the electrodes. To date, extensive studies have been made on carbon materials that are used as CDI electrodes [1,2,3,4,5,6,7,8,9,10,11,12]. Moreover, there are also studies based on the use of carbon nanotubes (CNTs) as CDI electrodes because of their exceptionally affordable functionality, structural stability, high surface area, and low electrical resistivity [13,14,15,16].

In a study, CNTs were used as electrodes in CDI systems, where the data obtained through experiments agree well when compared with the Langmuir model, and the highest desalination capacity was found to be 40 mg/g [17]. The desalination capacity was improved by compositing CNTs with polyaniline (PAni) [18]. The electrosorption capacity of CNTs/PAni electrodes was found to be higher than the electrode made from only SWCNTs, where the electrodes based on composite materials were regenerated, indicating excellent recyclability. CNT materials were also composited with graphene to increase the performance of the CDI system, as reported by Zhang et al. [19]. The observed excellent desalination behaviour of the composite electrodes is due to their higher electrical conductivity and surface area. The above-mentioned CNT-based electrodes were made as two-dimensional (2D) electrodes. Hence, it is necessary to develop CNT-based three-dimensional (3D) electrodes for better effectiveness in a CDI system because of their highly porous surface area and large amount of ion diffusion [20].

One important material that is used in 3D electrodes is reticulated vitreous carbon (RVC), which does not possess a significant surface area compared to several other carbons, like activated carbon (AC), activated carbon nanofibre (ACF) web [21], carbon nanotubes (CNT) [22], composite carbon nanotubes with carbon nanofibre (CNT/CNF) [23], reduced graphite oxide (rGO) [24], and carbon aerogels (CA) [25], which are used as 3D CDI electrode materials. Hence, RVC can be used as a 3D template for all other materials because of its excellent properties, such as the reduction in the resistance of solution flow through the electrode, the increase in the stability of composite electrodes towards high flow-rate pressure, the increase in the possibility of ions to reach all electrode surfaces in a short time for electrosorption, and the reduction in the time to release the ions from the electrode surface, compared to the other 3D electrodes.

It is clear from the above discussion that studies on the development of composite electrodes in CDI systems based on RVC and CNTs is very rare. Previously, we reported how the 3D poly(3,4-ethylenedioxythiophene/reticulated vitreous carbon PEDOT/RVC electrodes [26,27] can be used efficiently in CDI systems where RVC was used to support PEDOT. This motivated us to use RVC electrodes to build 3D nanoweb-functionalized SWCNT electrode structures for CDI systems by coating RVC with SWCNT using a dip-coating methodology.

The objective of the present study is to prepare RVC/SWCNT electrodes and use them in the CDI system for desalination. In this study, acid functionalization of single-wall carbon nanotubes (a-SWCNTs) was done and then coated on the RVC electrode, which was then used as a 3D template/support. Actually, the pristine CNTs have a tendency to form aggregates within the composite system. Hence, to avoid agglomeration and to obtain better dispersion, the CNTs were functionalized. This is because the functional group will keep the CNT particles apart from each other by preventing the formation of aggregates. It also helps to form the porous structure. Moreover, functionalization converts CNTs into hydrophilic materials that reduce the flow resistance of aqueous solutions through the electrodes. These electrodes were tested in a capacitive deionization system and the performance of these electrodes in the system was investigated under various working conditions, such as the flow rate and bias potential, which were optimized. Moreover, the effect of the feed stream directly through the electrode or between the electrodes, and the distance between the electrodes in the CDI system, was investigated. Furthermore, the interaction of ions with the electrodes was investigated using the electrosorption isotherms, such as Langmuir and Freundlich models, and the performance of the electrodes was evaluated by electrosorption dynamics. The basic and improved design of flow-through cells made by 3D printing was also considered. All of these are important for developing electrodes and to use effectively in purification technology.

## 2. Materials, Methods, and Experiments

### 2.1. Chemicals and Materials

SWCNTs (Hipco-CCNI/Lot#p1001) were supplied by Carbon Nanotechnologies, Inc. (Houston, TX, USA) and were used as received. The solvent, *N*,*N*-dimethylformamide (DMF) (AR grade), concentrated HNO_3_ (70%), and NaCl (AR grade) were purchased from Sigma-Aldrich (Darmstadt, Germany) and were used as received. The RVC (compressed 60 ppi (pores per inch)), procured from ERG Materials and Aerospace Engineering (Oakland, CA, USA), was cleaned before use. Membrane filters (0.2 µm GTTP) were supplied by Millipore (Sigma-Aldrich Darmstadt, Germany). Milli-Q water possessing a resistivity of 18.2 MΩ cm was used in all preparations.

### 2.2. Method to Functionalize Carbon Nanotubes

The functionalization of SWCNTs (20 mg dispersed in 40 mL 6 M HNO_3_) was carried out by refluxing in a round-bottom flask fitted with a reflux condenser and a magnetic stirrer. The reflux was performed by placing the flask in an oil bath at 120 °C for 6 h. The product was then filtered and washed with water until neutralization, and subsequently washed with methanol (10 mL) and DMF (10 mL), and then dried in an oven for 48 h at 105 °C. The acid-treated SWCNTs were designated as a-SWCNTs.

### 2.3. Pre-Treatment of the RVC Electrodes

Before treatment, all the RVC electrodes were cut into pieces of equal dimension using an RVC block. The dimension of each piece was 4 cm × 1.8 cm × 0.3 cm (length × width × thickness) = 2.16 cm^3^. Initially, the electrodes were cleaned to remove impurities from their surfaces by soaking in 2 M HNO_3_ for 24 h and then the acid was neutralized by washing several times with distilled water. Then the electrodes were soaked further in methanol for 2 h to remove any other type of organic impurities. The electrodes were then dried by flowing nitrogen and heating overnight at 110 °C. The weight of the electrodes was noted accordingly.

### 2.4. Method of a-SWCNT Dip Coating on RVC Electrodes

Initially, for dip coating, all the treated RVC electrodes were immersed into the 0.2% *w*/*v* a-SWCNT solution. The immersion was done slowly to allow the escape of air bubbles and prevent the formation of air pockets. After immersion, within a few seconds, the whole part of the RVC electrode was filled with solution due to capillary action. The electrodes were removed and dried initially at room temperature overnight, then at 100 °C for 2 h in an oven, and finally at 50 °C for 2 h using a vacuum oven to remove all organic solvents from the micropores of the electrodes. To achieve maximum loading of a-SWCNT on RVC, the process was repeated 2, 5, 10, and 20 times. The quantity of a-SWCNT loading on the RVC substrate was determined by weighing the electrode before and after dip coating. The loadings of a-SWCNT onto the RVC substrate were 6 mg (3.63 wt%), 23 mg (12.50 wt%), 34 mg (17.43 wt%), and 50 mg (23.85 wt%), when the process was repeated 2, 5, 10, and 20 times, respectively. The process of dip coating is schematically shown in Figure S1 (see Supplementary Section S1).

### 2.5. Electrochemical and Microscopic Characterisation

Cyclic voltammetry (CV) was used to determine the capacitance of the electrodes. The composite electrode, a-SWCNT/RVC, acted as a working electrode (WE) in 1 M aqueous NaCl solution. The scanning was done using a three-electrode system within the voltage range −0.2 to 1.0 V and a scan rate of 5–200 mV/s. In this measurement, RVC was used as a counter electrode (CE) and Ag/AgCl (3 M NaCl) as a reference electrode (RE). The electrical contact between the WE and the CE was made by the use of Pt wire. The procedure for measuring the amount of ion removal from the NaCl aqueous solution and on how to construct a CDI cell has been described in Supplementary Sections S2 and S3 along with Figures S2 and S3, respectively. The morphology of the a-SWCNT-coated RVC electrodes was analyzed by using a field emission scanning electron microscope (FESEM, ZEISS Sigma, Hamburg, Germany) at a specific voltage of 0.5 kV.

### 2.6. Different Working Conditions for Measuring Ion Removal Efficiency

The performance of ion removal of NaCl onto the sites of a-SWCNT is greatly affected by different working conditions, like flow rate, applied electrical voltage, loading level of a-SWCNT, and cell configuration. A systematic investigation of these different conditions on the efficiency of ion removal has been carried out in this section. These experiments were carried out with 60 mL NaCl solution. The concentration of NaCl was 75 mg/L whose measured electrical conductivity was 143.00 μS/cm. In this measurement, RVC was used as a counter electrode and the Ag/AgCl electrode as a reference electrode. The temperature was kept constant at 293 K during the experiment.

### 2.7. CDI Cell Configuration and Its Salient Features

**Figure 1:**
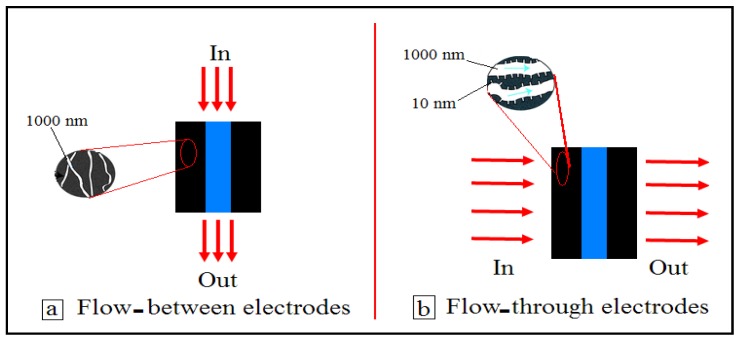
Schematic diagram of flow-between and flow-through electrodes in CDI cells.

While designing a CDI system, the fact that the feed stream to be desalinated should flow primarily between the two porous electrodes is considered [19,28,29,30]. The separation between the electrodes would be such that it acts as a flow channel and also prevent the electrical short circuit between the electrodes. Flow between (FB) electrodes in CDI systems require long desalination times [31]. Therefore, an attempt was made to change the location of the electrodes so that there is direct flow of the feed stream through the electrode pores to reduce the desalination time. This flow system, now called the flow-through (FT) electrode cell, often uses low hydraulic resistance and high surface area porous electrodes that consist of micropores and nanopores [31]. According to Suss et al. [31], when carbon aerogel is used in an FT cell, the mean sorption rate increases 4–10 times higher than that typically achieved with an FB cell. This is because the FT cell allows ion transport from the microscale pore bulk to the nanoscale pores, which maximizes the surface area [31]. Figure 1a,b represents the configuration of a typical FB charging electrode through a porous separator element and an FT capacitive desalination cell that allows the feed solution to flow directly through the electrode pores, respectively. The electrosorption performance of both cells was compared.

### 2.8. Working Conditions for Investigating the CDI Cell Configuration on Ion Removal Efficiency

In all the experiments 60 mL of the 75 mg/L NaCl solution was used and it was re-circulated at 50 mL/min as a feed solution in the CDI system under an applied voltage of 1.5 V. An a-SWCNT 50 mg coated RVC electrode (23.58 wt%) was used as the working electrode. Additionally, the distance between the electrodes in both configurations was 5 mm.

### 2.9. Designing a New Flow-Through Cell

**Figure 2:**
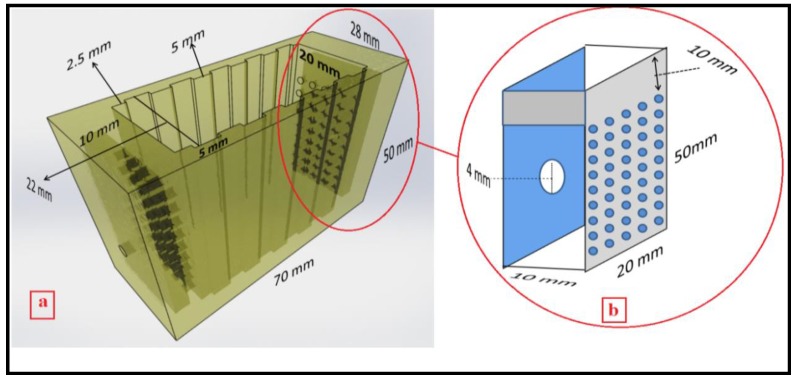
Schematic of a flow-through cell (**a**) and cross-section of flow-distributor chamber (**b**).

A new CDI cell was designed based on the flow-through electrode configuration aiming for obtaining a faster desorption cycle. Figure 2 shows the schematic of a new design made in our laboratory. It was built on a Connex 350 3D printer by Objet. This time the flow-through cell was produced using the MED610 material printed over a period of 3 h. This cell was rectangular shaped outside and the dimensions were 50 mm × 70 mm × 28 mm in height, length, and width, respectively (Figure 2a). Each side had one 4 mm diameter hole in the middle that served as the inlet and outlet ports for re-circulating the fluid flow by pumping. When entering the cell, the solution was passed through a flow distributor chamber 40 mm × 20 mm × 10 mm in height, length, and width, respectively, that had 45 (0.35 mm diameter) holes in the exit side that helped to direct flow onto the whole electrode surface (Figure 2b). A similar chamber was constructed for the outlet end. The cell was designed inside to fit a series of electrodes and maintain separation between them. The dimensions of each location and each separating space were 50 mm × 22 mm × 5 mm and 50 mm × 20 mm × 5 mm in height, length and width, respectively. This flow-through cell can hold up to five electrodes at any given time. The total volume of the solution was increased in this cell to 70 mL while the old cell was 60 mL. It is important to mention that the volume of the solution is a parameter in the electrosorption capacity equations (see Equations (S2)–(S4), Supplementary Section S4).

## 3. Results and Discussion

### 3.1. Scanning Electron Microscopy (SEM) and Cyclic Voltametry (CV)

The details of SEM and CV studies for RVC and CNT/RVC composites are presented in Supplementary Sections S5–S9. SEM was used to observe the morphology and calculate the pore size of RVC and CNT/RVC composite electrodes (shown in Figures S4 and S5). It was shown that the average pore size was 350, 700, and 900 µm for RVC electrodes having porosities 60, 45, and 30 ppi, respectively. The RVC electrode, containing a porosity of 60 ppi, was selected as the optimum electrode for loading the a-SWCNT because it showed the highest specific capacitance value and surface area per volume of the electrode. It was revealed that the pore was of the macroscale, through which the ions can diffuse easily. The specific capacitance was calculated through cyclic voltammetry (shown in Figures S6 and S7, and Table S1). It was increased by a factor of 280, 510, and 655 for 3.63, 12.50, and 17.43 wt% a-SWCNT/RVC electrodes, respectively, compared to a bare RVC electrode. The specific capacitance value was observed to gradually decrease with the increase in scan rate when tested for the 3.63 wt% a-SWCNT/RVC electrode. Moreover, the specific capacitance value, when measured per geometric volume, was seen to increase with the increase in the amount of a-SWCNT coating on RVC, indicating the increment in the coated surface area of a-SWCNT on the RVC electrode.

### 3.2. Effect of Applied Voltage on Ion Removal Efficiency

**Figure 3:**
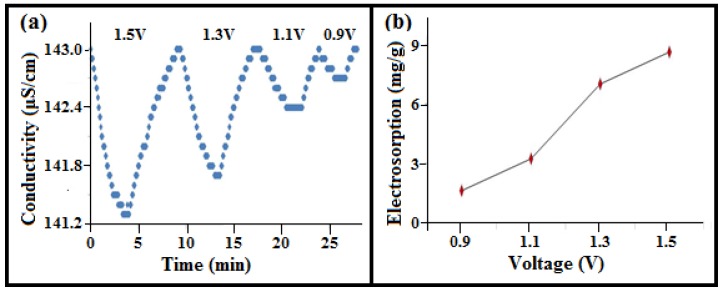
(**a**) Conductivity variations of NaCl solution in the adsorption and release time for 3.52 wt% a-SWCNT-coated RVC electrodes at a flow-rate of 25 mL/min with various applied voltages and operating times; and (**b**) electrosorption as a function of applied voltage.

In this study, the 3.63 wt% loaded a-SWCNT/RVC composite acted as a working electrode in the CDI system. The experiment was carried out within a voltage range of 0.9–1.5 V. The choice of this voltage range is based on some previous studies so that we can compare our results with the results reported in the literature [21,22,23,24,25]. The comparison has been made in the last section of this article. The variation in electrical conductivity of the NaCl solution with respect to the mentioned voltage range and operation time is shown in Figure 3a, whereas Figure 3b shows the variation of electrosorption capacity (calculated as explained in Supplementary Section S4) against the electrical voltage. The ion removal characteristics were affected by various applied voltages. With the applied electric field, there is a dramatic drop of salt concentration because the ions present in the system feel attraction by oppositely-charged electrodes [32]. As the applied voltage increased in the range of 0.9–1.5 V, the ion removal amount increased and the electrosorption capacity gradually rose from 3.15 mg/g to 8.92 mg/g. This clearly indicates that the electrosorption capacity increases with the increase in applied voltage, and this can be attributed to the strong Coulombic interaction originating between the electrodes and charged Na^+^ and Cl^−^ ions [24,33]. Electrolysis of water was also not observed at and above 1.2 V, as evident from the non-visibility/non-appearance of any gas bubbles from the solution, which can account for the existing resistance within the whole circuit [34,35]. It is observed from the plot that the electrical conductivity of the NaCl solution decreases approximately to 141.34 μS/cm, 141.61 μS/cm, 142.43 μS/cm, and 142.67 μS/cm at an applied voltage of 1.5 V, 1.3 V, 1.1 V, and 0.9 V, respectively. The plot also suggests that an efficient CDI process occurs at 1.5 V due to the enhancement of electrostatic force, whereas poor performance is observed at 1.1 V. When there is no variation in conductivity, it indicates that the ions were adsorbed maximally [32]. The electrosorption phenomenon exhibits its reversibility, as is evident after switching off the electric field, which results in the returning of conductivity to its initial state. Thus, it can be inferred that electrosorption processes in CDI cells and applied voltage are inter-related phenomena.

### 3.3. Role of the Flow-Rate on Electrosorption

**Figure 4:**
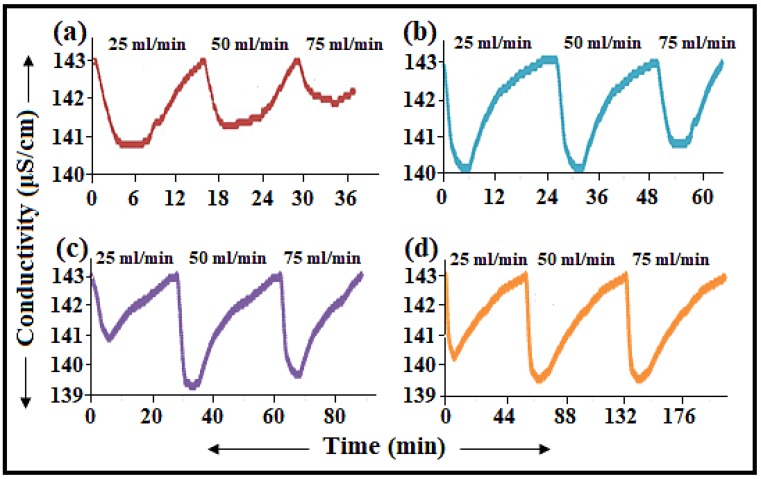
Variations in the conductivity of in the adsorption and release of NaCl solution at different applied flow rates and operating times when measured at 1.5 V: (**a**) 3.63 wt%; (**b**) 12.50 wt%; (**c**) 17.43 wt%; and (**d**) 23.58 wt% a-SWCNT-coated RVC electrodes.

The effect of increasing the flow-rate on the electrode’s ability to adsorb ions was investigated over a flow rate of 25–75 mL/min through a CDI cell as depicted in Figure 4. It is obvious that the efficiency of the removal of NaCl ions using 3.63 wt% a-SWCNT and 12.50 wt% a-SWCNT coating RVC electrodes decreases after increasing the flow rate above 50 mL/min as shown in Figure 4a,b. The maximum decrease in conductivity of the NaCl from an initial value of 143 μS/cm, using 3.63 wt% and 12.50 wt% a-SWCNT/RVC electrodes, is around 140.72 μS/cm and 140.14 μS/cm at a flow-rate of 25 and 50 mL/min, respectively. The conductivity at a flow rate of 75 mL/min was decreased to 141.33 and 140.82 μS/cm using 3.63 wt% and 12.50 wt% a-SWCNT/RVC electrodes, respectively. These results indicate that the flow rates above 50 mL/min would lead to lower electrosorption. This is because a high pump rate will exert a high pump force and if this force becomes greater than the electrosorption force then there will be decrement in the amount of electrosorption [28,34]. In addition, the conductivity characteristics related to flow rates were not significantly changed after the increment in flow rate from 25 to 50 mL/min. This is because the electrostatic force of the electrode reaches the equilibrium condition with the driving force of the flow rate [28,34]. Therefore, the amount of electrosorption did not significantly change in both cases. It is clear that when the loading level of a-SWCNT increased in the RVC electrodes to 17.43 wt% and 23.58 wt% (Figure 4c,d), the efficiency of the conductivity at the low flow rate (25 mL/min) was worse. The reason is that a low pump rate results in a co-ion effect that leads to depression of the electrosorption process [28,34]. Furthermore, the result shows that 50 mL/min is the optimal flow rate, as shown in Figure 4c,d.

Thus, it is evident that a voltage of 1.5 V and a flow rate of 50 mL/min are the optimum parameters exerting a positive effect on the ion removal performance of NaCl onto the sites of a-SWCNTs. Hence, for investigating the role of a-SWCNT loading on NaCl ion removal efficiency, these two parameters were taken as optimum conditions as discussed and reported in the later section of this article.

### 3.4. Effect of a-SWCNT Loading on Electrosorption

**Figure 5:**
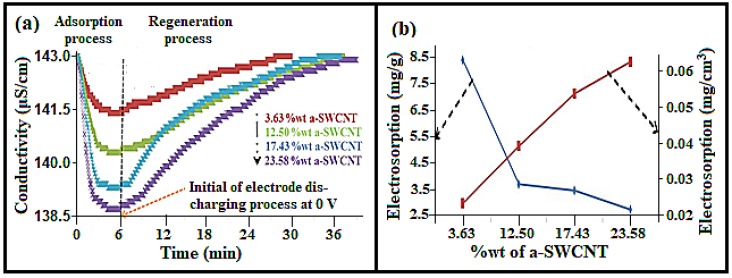
(**a**) Adsorption and release behaviour of various a-SWCNT-coated RVC electrodes at 1.5 volt, using 50 mL/min water flow-rate between electrode; and (**b**) the electrosorption capacity in terms of mass of a-SWCNTs and in terms of the geometric volume of the composite electrode at various loadings of a-SWCNTs on the RVC electrode using the CDI system.

The effect of increasing a-SWCNT loading on a-SWCNT/RVC composite electrodes on the ion removal performance was investigated at the loading levels of 3.63, 12.50, 17.43, and 23.58 wt%, respectively, and is depicted in Figure 5a. We choose these arbitrary loading levels to investigate the loading levels of SWCNTs on the electrosorption capacity, as well as to check the effectiveness of our electrodes compared to some published literature [21,22,23,24,25]. This has been discussed in the last section of this article. The figure shows that the application of electrical voltage decreases the conductivity of all electrodes because ions were attracted by opposite charges on the electrodes [32]. Then the conductivity approaches a minimum value, which implies that the system is saturated. These adsorption processes, at first, took 6 min. It is clear that the drop in conductivity of the solution increases with the increase in the amount of a-SWCNTs on the RVC electrode. This is because of the increment in loading of a-SWCNTs, which results in the increase in interaction between the charged electrode surfaces and Na^+^ and Cl^−^ ions. Notably, the highest drop in conductivity was 4.41 μS/cm using the electrode, which had 23.58 wt% a-SWCNT. For other electrodes, the conductivity decreased from 143.00 μS/cm to 141.34 μS/cm, 140.32 μS/cm, and 139.29 μS/cm with a-SWCNT loadings of 3.63 wt%, 12.50 wt%, and 17.43 wt%, respectively. Moreover, when the voltage of the CDI system is reduced to 0 V, the electrodes are found to be quickly regenerative. This indicates that the adsorbed ions on the electrodes are desorbed because of the disappeared electrostatic force.

It had taken almost 30 min to discharge all the ions from the electrode and to reach the conductivity at its initial state. Hence, it can be said that the CDI system made using the a-SWCNT/RVC composite can be effectively used for desalination technology.

The electrosorption removal of NaCl by our CDI system was measured from the data in Figure 5a. Hence, a calibration was made to find a relationship between conductivity (μS/cm) and concentration (mg/L) before conducting the experiment (see Equation (S1), Supplementary Section). The electrosorption behaviour of a-SWCNT-coated RVC electrodes is presented in Figure 5b, which shows that the electrosorption decreases with the increase in mass of a-SWCNT. It is clear that when the mass of a-SWCNT in 3.63 wt% a-SWCNT/RVC and 23.58 wt% a-SWCNT/RVC electrodes was 6 mg and 50 mg, the electrosorption was 8.39 mg/g and 2.77 mg/g, respectively. On the other hand, the value of electrosorption increases with the increase in the amount of a-SWCNT when the electrosorption of the electrode is considered in terms of the geometric volume or geometric area of the a-SWCNT coated on RVC. It is estimated that when the mass of a-SWCNT in 3.63 wt% a-SWCNT/RVC and 23.58 wt% a-SWCNT/RVC electrodes was 6 mg and 50 mg, the electrosorption was 0.003 mg/cm^2^ or 0.02 mg/cm^3^, and 0.008 mg/cm^2^ or 0.06 mg/cm^3^, respectively. Additionally, it is clear that the electrosorption became more stable above an electrode loading of 12.50 wt% a-SWCNT. Table 1 shows more details of the electrosorption in terms of mass, area, and volume for each a-SWCNT-coated RVC electrode. Hence, it can be inferred that the electrosorption performance of the 3.63 wt% a-SWCNT/RVC electrode is the best in terms of mg/g of ion removal, whereas the electrode with 23.58 wt% a-SWCNT loading exhibits the best ion removal efficiency in terms of either geometric area or geometric volume of the electrode. In other words, it can be said that the removal of ions is more dependent on the electrode size, which is a great advantage when one considers the size of the electrode to design a CDI system. Having designed a simple CDI system and investigated its electrosorption performance, attention was then turned towards refining the CDI system with an improved cell design. This work is now reported in the next section.

### 3.5. Effect of CDI Cell Configuration on the Removal Efficiency of Ions

**Figure 6:**
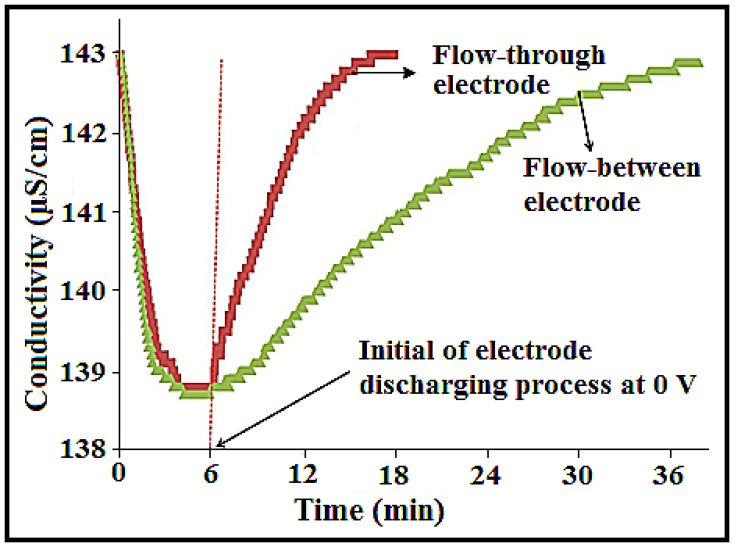
Comparison of NaCl adsorption and the release behaviour of the 23.58 wt% a-SWCNT-coated RVC electrode when configured for flow-between and flow-through systems in a CDI cell measured at a flow rate of 50 mL/min, 75 ppm NaCl, and 1.5 V.

Figure 6 shows the effect of feed stream for the flow-through (FT) electrode and the flow-between (FB) electrode with respect to the desalination time for the 23.58 wt% a-SWCNT-coated RVC electrode measured at a flow rate of 50 mL/min and a voltage of 1.5 V. It is observed that, to complete one desalination cycle, it takes 39 min and 18 min when the flow-between electrode and flow-through electrode configurations are used, respectively. In fact, the time required for desalination decreased more than two times when the flow-through electrode configuration was applied. In both systems, the adsorption process took 6 min, and the rest of the time was required to regenerate the electrodes. This means that the release of salt from the electrode required 12 min and 33 min for flow-through electrodes and flow-between electrodes, respectively. This improvement in the salt removal and regeneration process is due to the facilitation in ion transport at the electrode solution interface and faster electron transport within the electrode that resulted in the three times faster desalination cycle when the solution was flowed directly through the electrodes. It is very important to draw attention to the shape of the conductivity curve, although the amount of conductivity decrease was the same in both systems. The conductivity after 6 min in both systems was 138.59 μS/cm. This can be attributed to the flowing force of ion transport to the electrode interface, which was the same in both systems.

### 3.6. Effect of the Flow Rate and Voltage on Ion Removal Efficiency

**Figure 7:**
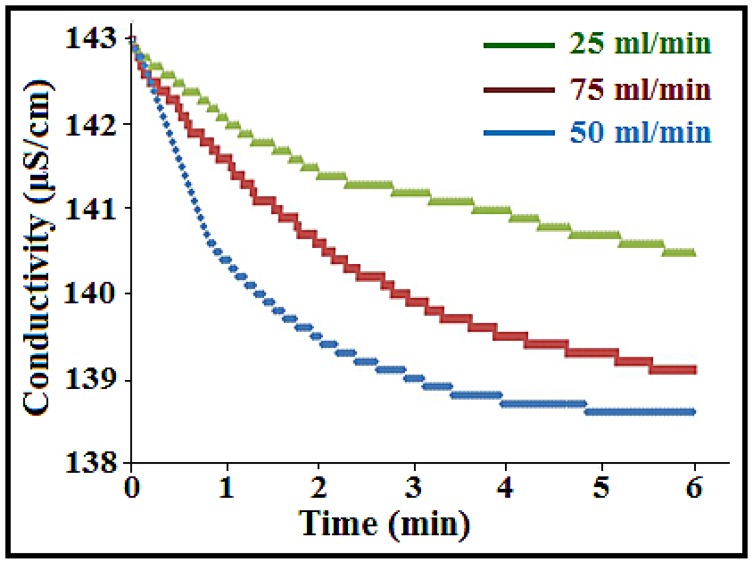
Conductivity variations of the NaCl solution for the 23.58 wt% a-SWCNT-coated RVC electrode as a function of operation time with respect to various flow rates (25 mL/min, 50 mL/min, and 75 mL/min) measured at 1.5 V.

In the new cell system, the total volume of solution was increased to 70 mL. The concentration and the electrical voltage were similar to the previous experiments (75 mg/L NaCl solute ion and 1.5 V). The flow rate was investigated to study the NaCl removal using the best performing electrode in the old system, which was the 23.58 wt% a-SWCNT-coated RVC electrode. The distance between the electrodes was 5 mm. According to the old system, the best performance for water purification was at a flow rate of 50 mL/min. This led to the investigation of electrode performance at flow rates below and above 50 mL/min, as shown in Figure 7. It is obvious from the results that the best conductivity decrease was achieved when the flow rate was at 50 mL/min. When the flow rate was above or below 50 mL/min, the conductivity decrease was less, leading to lower electrosorption capacity. This depression in electrosorption capacity below a flow rate of 50 mL/min is because of the co-ion effect resulting from a low pump rate, while above 50 mL/min, the pump force is higher than the electrosorption force at a high pump rate, thereby decreasing the amount of electrosorption [34]. Thus, the optimum flow rate for the CDI process was found to be 50 mL/min.

In the later sections, all experiments have been carried out with 70 mL of the 75 mg/L NaCl solution at a flow rate of 50 mL/min through the CDI system at an electrical voltage of 1.5 V.

### 3.7. Effect of the Distance between the Electrodes on the Efficiency of Electrosorption

**Figure 8:**
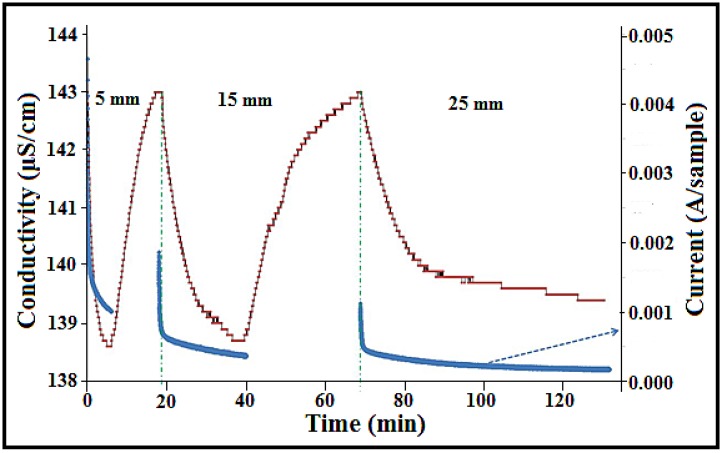
The effect of distance between the electrodes (5 mm, 15 mm, and 25 mm) on the current and conductivity behaviour of the 23.58 wt% a-SWCNT-coated RVC electrode measured at a voltage of 1.5 V and a flow rate of 50 mL/min.

It is known that increasing the space between a pair of electrodes results in an increase in electrical resistance between the electrodes that, in turn, leads to a decrease in the electrical current [36,37]. Therefore, the next phase of the study was directed towards the effect of current density and space between the electrodes on the efficiency of electrosorption. The space between the electrodes was varied from 5 mm to 15 mm and then to 25 mm, respectively, the counter electrode was the RVC electrode, and the working electrode was the 23.58 wt% a-SWCNT-coated RVC electrode. Figure 8 shows the current and conductivity behaviour of the 23.58 wt% a-SWCNT/RVC composite electrodes at various gaps between the electrodes. As expected, the current density decreased with an increase in the gap between the electrodes. When the distance between the electrodes was 5, 15, and 25 mm, the current at the start was found to be 4.56, 2.20, and 1.11 mA, respectively (Table 2). Table 2 also gives more details about the start current, current stability, charge of the electrode, the time of highest conductivity, and the energy output for each distance between the electrodes. Furthermore, there is a variation in the output energy of the CDI system when the distance between the electrodes is increased. It is clear that if the space increases, the energy output increases. The energy output at 5, 15, and 25 mm was 0.67, 0.91, and 1.42 J/C, respectively. The energy is calculated according to Equation (1) [38]:

*E* = *Q* × *V* (1)

where *E* is the energy (J), *Q* is the charge (C), and *V* is the constant voltage (*V*).

It is very interesting to note that the performance of ion removal is affected when the space between the electrodes is varied. With the increase in distance between the electrodes, the amount of ions removed was not affected in the range of distances from 5 mm to 15 mm, but was less affected when the gap between the electrodes was 25 mm. However, there is an increment in the adsorption time as the distance increases in all cases. One deionisation cycle took 7 min at a distance of 5 mm between the electrodes, but when the distance was increased to 15 mm the ion adsorption/desorption cycle took 55 min, as shown in Figure 8. As mentioned above, the best design should have a small space between the electrodes because this will reduce the time of water purification, afford efficient energy output, decrease the electrical resistance between the electrodes, and increase the electrical current.

### 3.8. Adsorption/Desorption Performance of a-SWCNT Loaded RVC Electrodes

**Figure 9:**
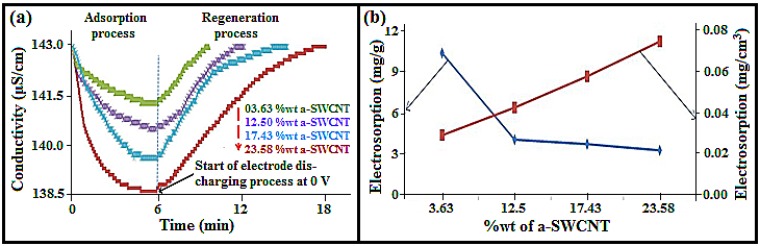
(**a**) Adsorption and release behaviour; and (**b**) the electrosorption capacity in terms of the mass of a-SWCNT and the geometric volume of the electrode at various a-SWCNT-coated RVC electrode coatings of a-SWCNT (wt%): 3.63%, 12.50%, 17.43%, and 23.58%.

Figure 9a shows the CDI process of adsorption and desorption performance at all loading levels of a-SWCNT in RVC electrodes experimented at an optimized electrode distance, electrical voltage, and flow rate. It is clear that the electrosorption behaviours of the electrodes measured using the new cell followed the same trend like the old cell, as mentioned earlier; that is, the drop in conductivity increases with the increase in the amount of a-SWCNT on the electrodes. It is noticed that for the 23.58 wt% a-SWCNT-coated RVC electrode, there is a decrease of about 4.41 μS/cm in electrical conductivity, indicating a significant achievement in electrosorption performance. Initially, for all electrodes, the conductivity gradually decreases up to a minimum value, which indicates that saturation was reached. These adsorption processes took 6 min. During discharging of the CDI system at 0 V, the conductivity returned nearly to its initial state (143 μS/cm). This is an indication to the release of ions from their electrical double-layer region back into the solution because of the disappearance of electrostatic forces, as have been discussed earlier. It took 18 min for complete discharge of the CDI cells for the electrode with the highest amount of a-SWCNT in the sample (23.58 wt%). This result is in agreement with the earlier reported results in Figure 5a.

Figure 9b shows the electrosorption behaviour of different a-SWCNT/RVC composite electrodes in terms of the mass of a-SWCNT and the volume of the electrode. It is clear that the electrosorption decreases with the increase in the weight of a-SWCNT loading. Clearly, when the sample had 3.63 wt% a-SWCNT, the electrosorption capacity was 10.40 mg/g and when the sample had 23.58 wt% a-SWCNT, the electrosorption capacity was 3.23 mg/g. On the other hand, if the electrosorption of electrodes was considered in terms of the geometric area or geometric volume, the electrosorption increased with the increase in the amount of a-SWCNT. For example, when the sample had 3.63 wt% a-SWCNT, the electrosorption was 0.03 mg/cm^3^ or 0.003 mg/cm^2^, and when the sample had 23.58 wt% a-SWCNT, the electrosorption was 0.08 mg/cm^3^ or 0.009 mg/cm^2^ (Table 3). Table 3 also gives more details about electrosorption in terms of mass, geometric area, and geometric volume for each a-SWCNT/RVC composite electrode.

### 3.9. Comparison between the New Cell (Flow Feed-Through Electrode) and the Old Cell (Flow Feed-Between Electrode)

This section compares the results of electrosorption capacity and the time of one desalination cycle, obtained with various a-SWCNT-coated RVC electrodes, for two different flow cells (flow feed-through electrodes and flow feed-between electrodes), as shown in Table 4. During the experiment, the concentration of the NaCl feed solution was 75 mg/L. Table 4 shows that the electrosorption capacity of electrodes has increased when measured using the new cell. The increment in elecrosorption capacity is from 8.39 mg/g to 10.40 mg/g, which is 23.96% (see example calculation below) if we consider the 3.63 wt% a-SWCNT/RVC composite electrode. In fact, there is approximately a three times decrement in the desalination cycle time, for example, from 30 min to 10 min, from 36 min to 12 min, from 37 min to 15 min, and from 39 min to 18 min when measured using the new cell for 3.63 wt% a-SWCNT, 12.50 wt% a-SWCNT, 17.43 wt% a-SWCNT, and 23.58 wt% a-SWCNT-coated RVC electrodes, respectively.

### 3.10. CDI Cycling Stability

**Figure 10:**
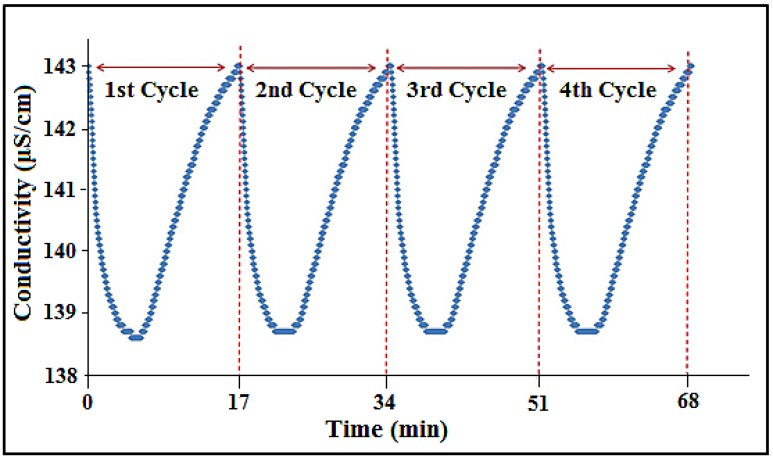
Multiple electrosorption–desorption cycles of 75 ppm NaCl solution for the 23.58 wt% a-SWCNT-coated RVC electrode. Polarization and depolarization were performed at 1.5 V and 0.0 V, respectively.

Regeneration of the a-SWCNT/RVC electrode is very important because of its practical applicability in CDI systems. The 23.58 wt% a-SWCNT electrode was used in this study because, previously, it exhibited the highest electrosorption capacity when measured per geometric volume using the flow feed-through cell. Figure 10 shows the electrosorption/regeneration cycles of the 23.58 wt% a-SWCNT-coated RVC electrode, which was performed repeatedly several times by charging and regeneration cycles. In the absence of the oxidation–reduction process in electrosorption, the consumption of current is basically to charge the electrode by electro-adsorbing the ions from the bulk of the solution [39]. It is seen from the figure that, initially, the solution conductivity when measured at 1.5 V sharply decreases because of the migration of ions onto the surfaces of oppositely-charged electrodes, and then there is a gradual decrement until the complete formation of an electrical double layer at the electrode/electrolyte interface [40]. The electrode is regenerated by depolarizing the system at 0.0 V. Herein, we have conducted four cycles of repeated electrosorption and desorption. It is observed that each cycle takes 17 min, which means 6 min to adsorb ions and 11 min to release ions, indicating that each cycle is going to complete in a short time period. Moreover, the stability of cycles is also high as there is no decay in electrosorption capacity. This reveals that the process is reversible and the composite electrode can be reused several times by controlling the amount of electro-adsorbed ions by manipulating the formed electrical double layer at the electrode/electrolyte interface.

### 3.11. Electrosorption Dynamics

**Figure 11:**
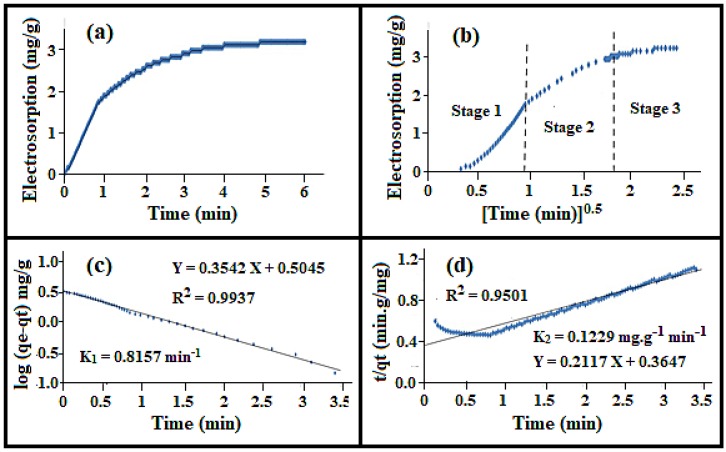
The (**a**) electrosorption; (**b**) intraparticle diffusion; (**c**) pseudo-first-order adsorption kinetics; and (**d**) pseudo-second-order adsorption kinetics of NaCl onto the 23.58 wt% a-SWCNT-coated RVC electrode at a flow rate of 50 mL/min and a voltage of 1.5 V.

The different electrosorption dynamics models for NaCl adsorption onto the 23.58 wt% a-SWCNT/RVC composite electrode measured at 1.5 V and a flow rate of 50 mL/min using a new cell are presented in Figure 11. The studied electrosorption dynamics models are pseudo-first-order, pseudo-second-order, and intra-particle diffusion dynamic models. The electrosorption of NaCl onto the electrode was very rapid within the first minute, then it became dynamic adsorption and after 3 min the electrode gradually approached saturation, as shown in Figure 11a. It takes 6 min to reach the electrosorption at equilibrium. This may be because of the high diffusion rate of ions onto the a-SWCNT particle surfaces. When the Weber and Morris model (see Equation (S9) in Supplementary Section S11) [41] is used to study the intra-particle diffusion of ions into the electrode, it is clear that the plot of *q*_t_ versus *t*^0.5^ shows three multi-linear regions, as shown in Figure 11b. This behaviour is quite common in an electrosorption process [28,34,42,43,44] and it indicates that more than one step is effective in the electrosorption process. When the voltage was applied, the first-stage adsorption occurred rapidly because the surface diffusion was transported from the bulk solution to the external surface of the sorbent [45]. In the second stage of adsorption, the adsorption occurs gradually in a linear manner. In this stage, the diffused intra-molecular sorbate molecules move more into the interior part of the sorbent particles. The third linear stage indicates that a low adsorption stage has occurred where intra-particle diffusion starts on the interior sites of the sorbent [45]. The intra-particle diffusion rate constant, *k*_id_, calculated from the linear slope from stage 2 and the coefficient of correlation are presented in Table 5 [46]. These two parameters are 1.66 mg/gmin^0.5^ and 0.981, respectively.

For evaluating the kinetics of the electrosorption process, the pseudo-first-order and pseudo-second-order processes were studied within the first 3 min, as shown in Figure 11c,d, respectively. The data of log (*q*_e_ − *q*_t_) were plotted against *t* where the slope gives the value of first-order rate constant *k*_1_ and the intercept represents the equilibrium adsorption density *q*_e_, as shown in Figure 11c. It is seen that the linear fit has a relatively high correlation coefficient (*R*^2^ value), suggesting that the application of Equation (S10) (see Supplementary Section S12) is appropriate as the plotted experimental data are in good agreement with their linear fit. The extracted parameters are reported in Table 5, which shows that the value of *R*^2^ and *k*_1_ for the pseudo-first-order kinetic model are 0.994 and 0.82 min^−1^, respectively. The obtained value for *q*_e_ (3.19 mg/g) is also reasonable according to this kinetic model.

To determine the adsorption parameters of the pseudo-second-order process, namely *q*_e_ and *k*_2_ as mentioned in Equation (S11) (see Supplementary Section S13), *t*/*q*_t_ was plotted against *t* as shown in Figure 11d. The figure shows that there is poor agreement between the experimental data and the theoretical values obtained using this model. The parameter values obtained from the second-order kinetic model are also tabulated in Table 5 and compared with the first-order kinetic model. The values of *R*^2^ and *k*_2_ for the second-order kinetic model obtained were 0.950 and 0.12 g/mg min, respectively, which revealed that the *R*^2^ value in the second-order kinetic model is poor compared to the first-order kinetic model (0.996). The calculated *q_e_* value is also not in good agreement with the experimental data (it is 4.72 mg/g). The above results suggest that the NaCl adsorption process follows the first-order kinetic model and confirms that the ions are not adsorbed onto the surfaces of a-SWCNTs via chemical interaction [45]. Many authors have reported a similar type of result in the past where NaCl ions were adsorbed from the aqueous solution by different adsorbents [7,24,28,34,42,43,44].

### 3.12. Electrosorption Isotherms

**Figure 12:**
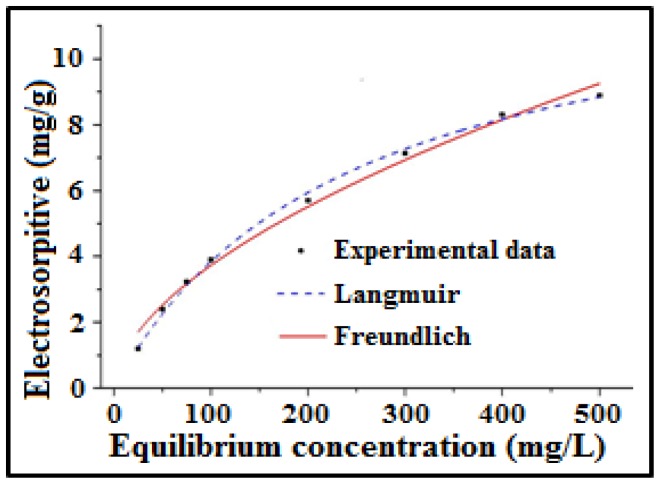
The electrosorption isotherm for the 23.58 wt% a-SWCNT-coated RVC electrode at a flow rate of 50 mL/min NaCl and a voltage of 1.5 V.

Electrosorption isotherms are essential to estimate the electrosorption behaviour of carbon electrodes. The electrosorption isotherm of NaCl was experimented onto the a-SWCNT (23.58 wt%)-coated RVC electrode at a voltage of 1.5 V, a flow-rate of 50 mL/min, and a temperature of 298 K using the CDI unit cell at different NaCl solution concentrations, starting from 25 mg/L to 500 mg/L, as shown in Figure 12. The electrosorption of a-SWCNT was affected at different initial solution concentrations. The figure shows that the increase in the solution feed concentration increases the removal of NaCl, which means the increase in electrosorption behaviour. This is because of the enhancement of the mass transfer rate of ions within the micropores and the subsequent reduction in the overlapping effect due to the increment in the concentration of solution [43,47,48]. It is noticed that at a feed solution concentration of 500 mg/L, the electrosorption capacity of the 23.58 wt% SWCNT-coated RVC electrode is 8.89 mg/g.

The experimental results were fitted to Langmuir and Freundlich isotherms (Equations (S12) and (S13), respectively; see Supplementary Section S14) for evaluating the electrosorption behaviour of Na^+^ and Cl^−1^ onto the electrodes. Table 6 shows the value of the extracted parameters, such as the coefficients of correlation *R*^2^, the Langmuir constant *K*_L_ related to binding energy, and the Freundlich constant *K*_F_ related to the adsorption capacity of adsorbent for NaCl electrosorption using the 23.58 wt% a-SWCNT-coated RVC electrode as an electrosorption electrode. It is found that the plots of electrosorption obtained using both isotherm models are in good agreement with the experimental plot for this electrode, which is also evident from their *R*^2^ values, which is 0.997 for the Langmuir model and 0.989 for the Freundlich model. Thus, the monolayer adsorption can be suggested as the primary mechanism for this electrosorption adsorption process [7,28]. The *K*_L_ and *K*_F_ values of the a-SWCNT electrode were 0.01 and 0.28, respectively. Normally, for high adsorption, the value of *n*, which indicates the tendency of the adsorbate to be adsorbed should be between 1 and 10 [34]. The value of *n* in this electrode was 1.77, indicating that the a-SWCNT electrode exhibits a high potential electrosorption capability. One can obtain a very thin layer of adsorbate for such a system where the adsorbed amount will be a fraction of the monolayer capacity. Hence, the electrosorption of the a-SWCNT/RVC electrode in the CDI system follows the monolayer adsorption [42]. Moreover, for estimating the maximum electrosorption amount of a-SWCNT, the Langmuir isotherm model was selected. The parameter *q*_m_ in this model accounts for the maximum adsorption capacity, which suggested that *q*_m_ was improved as the bias concentration increase.The equilibrium electrosorption capacity of this electrode at the above mentioned experimental conditions was 13.08 mg/g.

### 3.13. Comparison of the Present Work with the Previously-Published Research Studies

In this section, the electrosorption capacities of a-SWCNT-coated RVC electrodes were compared with several other carbon materials, such as activated carbon (AC), activated carbon nanofibre (ACF) web [21], carbon nanotubes (CNT) [22], composite carbon nanotubes with carbon nanofibre (CNT/CNF) [23], reduced graphite oxide (rGO) [24], and carbon aerogels (CA) [25], which are used as CDI electrode materials. Table 7 lists the electrosorption capacity, mass, and dimensions of electrode materials, and other basic information of all previous carbon electrode materials for the CDI systems. It can be observed that all researchers measured the electrosorption capacity by mg of NaCl per gram of the active material and they ignored measuring it by the geometric area or geometric volume. This leads to the comparison of all electrosorption capacities by the weight of the activated carbon materials in the electrode. However, our research focussed on improving the electrosorption capacity in terms of the geometric area and geometric volume. It can be seen that 3.63 wt% of a-SWCNT-coated RVC gave the highest amount of electrosorption capacity (10.40 mg/g) than that of the other electrode materials. Additionally, the electrode that had 23.58 wt% a-SWCNT-coated RVC still had a high electrosorption capacity (3.23 mg/g) compared to the CNT electrode material (2.33 mg/g). It is worth mentioning that the time required for one adsorption cycle in the CNT electrode material, which was 100 min, was decreased more than 16 times when the a-SWCNT-coated RVC electrode was used (6 min). This means that the amount of pure water produced is increased more than 16 times when the a-SWCNT-coated RVC electrode is used.

The electrosorption capacity of the activated carbon nanofibre (ACF) web was consistently higher than that of AC, CA, CNT, CNT/CNF, rGO, and all a-SWCNT-coated RVC electrodes, except the 3.25 wt% SWCNT-coated RVC, as shown in Table 7. Unfortunately, this ACF electrode functioned with a very low flow rate (5 mL/min) and needed a long time to complete one cycle of adsorption (150 min). The main differences between the CDI electrodes include the energy consumed in the CDI system. It is known that the energy consumed increases with the increase in the time required for the adsorption processes and the increase in the cell voltage [38]. Table 7 shows the applied cell voltages and time for one adsorption cycle. It is noted that the applied cell voltage for all a-SWCNT-coated RVC electrodes was 1.5 V as for the AC, CA, and rGO electrodes. The differences between these electrodes were the time taken for one adsorption cycle (6 min for a-SWCNT-coated RVC electrode, 30 min for AC and rGO, and 840 min for CA). It is observed that the energy consumed in the CDI system with the a-SWCNT-coated RVC electrode is much less compared to the other electrodes. Additionally, a comparison of the time per adsorption cycle of a-SWCNT-coated RVC (6 min) with other electrodes in Table 7 shows that the time is much shorter, which leads to energy saving.

## 4. Conclusions

The construction of a unique 3D electrode based on a-SWCNT-coated RVC composites has been performed in this study and their electrochemical performance has been checked by cyclic voltammetry. The optimum voltage and flow rate for the electrodes were 1.5 V and 50 mL/min, respectively, at which the NaCl ion removal performance was mostly affected onto the sites of a-SWCNT. The effect of increasing a-SWCNT loading on the ion removal performance of a-SWCNT/RVC composite electrodes was investigated, which suggested that the composite electrodes can be effectively used in the CDI cell for desalination technology. The CDI system was tested for flow-between and flow-through electrode configurations. It is revealed that the flow-through electrode configuration takes 18 min for one desalination cycle, which means the desorption cycle is three times faster compared to the flow-between electrode configuration, which takes 54 min. A new CDI cell was designed based on the flow-through electrode configuration, which revealed that the electrosorption capacity for all electrodes was increased with the simultaneous reduction in desalination cycle time. The electrosorption capacity for the 3.63 wt% a-SWCNT-coated RVC electrode composite was increased by 23.96%, with a three times reduction in desalination cycle time when the flow-through electrode is used in a new CDI cell. It has been shown that when the distance between the two electrodes is increased, the adsorption time also increases. It is observed that the NaCl ion adsorption characteristics follow the pseudo-first-order kinetic model compared to the pseudo-second-order kinetic model. However, the electrosorption behavior of the 23.58 wt% a-SWCNT electrode is in good agreement with both the Langmuir and Freundlich isotherm models, suggesting monolayer adsorption as the primary mechanism in the electrosorption process. The maximum electrosorption capacity at the highest solution feed concentration was 8.89 mg/g, whereas the theoretical maximum value was 13.08 mg/g, as per the calculation using the Langmuir isotherm model. It has been shown that our developed electrode based on 3.63 wt% of a-SWCNT-coated RVC showed the highest amount of electrosorption capacity (10.40 mg/g) compared to the other electrode materials. Moreover, the time taken for one desalination cycle in the case of the CNT electrode materials (100 min) was more than 16 times less when our a-SWCNT-coated RVC electrode was used (6 min). This indicates that, within the same time period, the amount of pure water production increased more than 16 times when the a-SWCNT-coated RVC electrode is used. Additionally, the energy consumption in the CDI system with the a-SWCNT-coated RVC electrode is much less compared to the other electrodes.

## Figures and Tables

**Table 1:** Electrosorption capacity of various a-SWCNT-coated RVC electrodes.

Sample	a-SWCNT in Sample (wt%)	Electrosorption Capacity		
		mg/g of a-SWCNT	mg/cm^3^ of Electrode	mg/cm^2^ of Electrode
1	3.63	8.39	0.02	2.8 × 10^−3^
2	12.50	3.69	0.03	4.8 × 10^−3^
3	17.43	3.42	0.05	6.5 × 10^−3^
4	23.74	2.77	0.06	7.6 × 10^−3^

**Table 2:** The current of the CDI system for different inter-electrode distances using 1.5 V.

Distance between Electrodes (mm)	Time Required for Adsorption (min)	Initial Current (mA)	Stable Current (mA)	Charge (C)	Energy (J)
5	6	4.56	1.02	0.45	0.67
15	25	2.20	0.27	0.61	0.91
25	60	1.11	0.16	0.95	1.42

**Table 3:** Electrosorption capacity of different a-SWCNT/RVC composite electrodes.

Sample	a-SWCNT in Sample (wt%)	Electrosorption Capacity		
		mg/g of a-SWCNT	mg/cm^3^ of Electrode	mg/cm^2^ of Electrode
1	3.63	10.40	0.03	3.5 × 10^−3^
2	12.50	3.99	0.05	5.1 × 10^−3^
3	17.43	3.67	0.06	7.2 × 10^−3^
4	23.58	3.23	0.08	9.4 × 10^−3^

**Table 4:** Electrosorption capacities and the time of one desalination cycle of various a-SWCNT-coated RVC electrodes obtained by comparing the performance between flow feed-between (FB) electrodes and flow feed-through (FT) electrodes with the NaCl feed solution (75 mg/L).

a-SWCNT in the RVC Electrode (wt%)	Flow Direction	Electrosorption (mg/g of a-SWCNT)	# Enhancement in Electrosorption (%)	Time of One Desalination Cycle (min)
3.63	FT	10.40	23.96	10
	FB	8.39	-	30
12.50	FT	3.99	8.13	12
	FB	3.69	-	36
17.43	FT	3.67	7.31	15
	FB	3.42	-	37
23.58	FT	3.23	16.61	18
	FB	2.77	-	39

# An example calculation is given in Supplementary Section S10.

**Table 5:** Different parameter values obtained using the pseudo-first-order model, pseudo-second-order model, and intraparticle diffusion.

Pseudo-First-Order			Pseudo-Second-Order				Intraparticle Diffusion	
*q*_e_ (mg/g)	*K*_1_ (min^−1^)	*R* ^2^	*q*_e_ (mg/g)	*K*_2_ (g/mg min)	*R* ^2^	h (mg/g min)	*K*_id_ (mg/g min^0.5^)	*R* ^2^
3.19	0.82	0.994	4.72	0.123	0.950	2.74	1.66	0.981

**Table 6:** Determined parameters of regression coefficients *R*^2^, *K*_L_, and *K*_F_ of Langmuir and Freundlich isotherms for NaCl electrosorption by using the a-SWCNT (23.58 wt%)-coated RVC electrode as an electrosorption electrode.

Isotherm	Parameter	Value
Langmuir	*q*_m_	13.08
	*K*_L_	0.01
	*R* ^2^	0.997
Freundlich	*K*_F_	0.28
	*n*	1.77
	*R* ^2^	0.989

**Table 7:** Comparison of electrosorption capacity of various carbon electrodes and electrodes developed in this article.

Parameters	Carbon Electrodes						Electrodes Developed in This Article (SWCNT/RVC) Using a New Cell			
	AC	CA	CNT	CNT/CNF	rGO	ACF	3.63 wt%	12.50 wt%	17.43 wt%	23.58 wt%
Electrosorption capacity (mg/g)	1.51	2.56	2.33	3.32	3.23	4.64	10.40	3.99	3.67	3.23
Electrosorption Capacity (mg/cm^3^)	-	-	-	-	-	-	0.029	0.043	0.058	0.075
Electrosorption capacity (mg/cm^2^)	-	-	-	-	-	-	0.039	0.057	0.078	0.10
Mass of materials (g)	1.50	4.30	0.12	0.85	1.50	0.31	0.006	0.023	0.034	0.050
Electrode length and width (cm)	14 × 7	16 × 8	8 × 8	8.7 Diameter	14 × 7	7 × 5	4 × 1.8			
Electrode thickness (mm)	0.3	0.8	0.03	0.3	0.3	0.2	3.0			
Applied voltage (V)	1.5	1.5	1.2	1.2	1.5	1.6	1.5			
Flow rate (mL/min)	20	400	40	14	20	5	50			
Solution volume (mL)	200	500	50	40	200	50	70			
Initial conductivity of NaCl solution (µS/cm)	145	101	100	100	145	192	143			
Time of electrosorption cycle (min)	30	840	100	45	30	150	6			
Reference	[24]	[25]	[22]	[23]	[24]	[21]				

## Supplementary Materials

The following are available online at http://www.mdpi.com/2079-4991/8/7/527/s1. Figure S1: The full process of dip coating RVC in an a-SWCNT solution, Figure S2: Calibration curve linearity for ionic conductivity vs. NaCl concentration, Figure S3: Schematic diagram of a capacitive deionization (CDI) cell, Figure S4: (a–c) Photos and (d–f) SEM micrographs of 60, 45, and 30 ppi RVC samples, respectively; (g) the specific capacitance of a-SWCNT-coated RVC electrodes of various porosities in 1 M NaCl solution calculated from cyclic voltammograms recorded in a voltage range between −0.2 and 1.0 V using a three-electrode system vs. Ag/AgCl at a 5-mV/s scan rate, Figure S5: Scanning electron microscopy (SEM) images (a,b) top surface, (c) cross-section, and (d,e) 45° view of 23.58 wt% a-SWCNT loading, Figure S6: (a) Comparison cyclic voltammograms of the 1 cm^3^ bare RVC electrode and the same size of the 23.58 wt% a-SWCNT-coated RVC electrode in 1 M NaCl scanned at 20 mV/s and using an RVC counter electrode and Ag/AgCl reference electrode in a three-electrode system; and (b) the effect of a-SWCNT loading of a-SWCNT/RVC composite electrodes on specific capacitance, Figure S7: (a) Cyclic voltammograms of the 3.63 wt% a-SWCNT-coated RVC electrode and (b) specific capacitance of various a-SWCNT-coated RVC electrodes at various scan rates in 1 M NaCl solution in the voltage range between −0.2 and 1.0 V vs. Ag/AgCl using a three-electrode system. Cyclic voltammograms of a-SWCNT-coated RVC electrodes at 20 mV/s, in terms of (c) current per gram of a-SWCNT and (d) current per geometric volume of the electrode; and (e) capacitance of the electrodes per gram of a-SWCNT and per geometric volume of the electrode, Table S1: Specific capacitance of various a-SWCNT-coated RVC electrodes by F/g of a-SWCNT, F/area of the electrode, and F/volume of the electrode, in 1 M NaCl solution at various scan rates.
