# Supplementary material for: Single-Walled Carbon Nanotube (SWCNT) Loaded Porous Reticulated Vitreous Carbon (RVC) Electrodes Used in a Capacitive Deionization (CDI) Cell for Effective Desalination
Source: Nanomaterials (Basel). 2018 Jul 13;8(7):527. doi: 10.3390/nano8070527 (PMC6071079; doi:10.3390/nano8070527)
Supplement: Supplementary file 1 [file nanomaterials-08-00527-s001.pdf]

nanomaterials

Supporting Information

Single-Walled Carbon Nanotube (SWCNT) Loaded Porous Reticulated Vitreous Carbon (RVC) Electrodes Used in a Capacitive Deionization (CDI) Cell for Effective Desalination

Ali Aldalbahi [1,*], Mostafizur Rahaman [1], Mohammed Almoiqli [2], Abdelrazig Hamedelniel [1] and Abdulaziz Alrehaili [1]

S1. Schematic Diagram of a-SWCNT Dip Coated on RVC Electrode

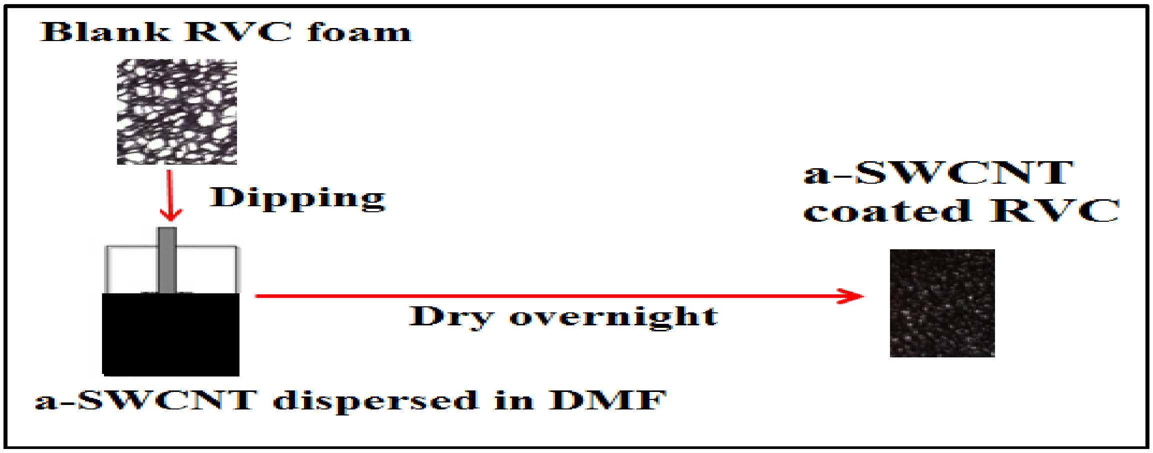

Figure S1. The full process of dip coating RVC in an a-SWCNT solution.

S2. Measurement and Calculation of Ion Removal from NaCl Aqueous Solution

Measurement of Ion Removal from NaCl Solution

NaCl concentration was determined in our laboratory by measuring the electrical conductivity of a NaCl solution. The calibration curve linearity is shown in Figure S2. Conductivity linearly increased as the NaCl concentration increased. The equation from fitting a line starting from the origin (0,0) to the calibration curve is as follows;

$$Cond = 1.9067 \times Conc \quad (S1)$$

where *Cond* and *Conc* are the conductivity (μS/cm) and concentration (mg/L) of the NaCl solution, respectively.

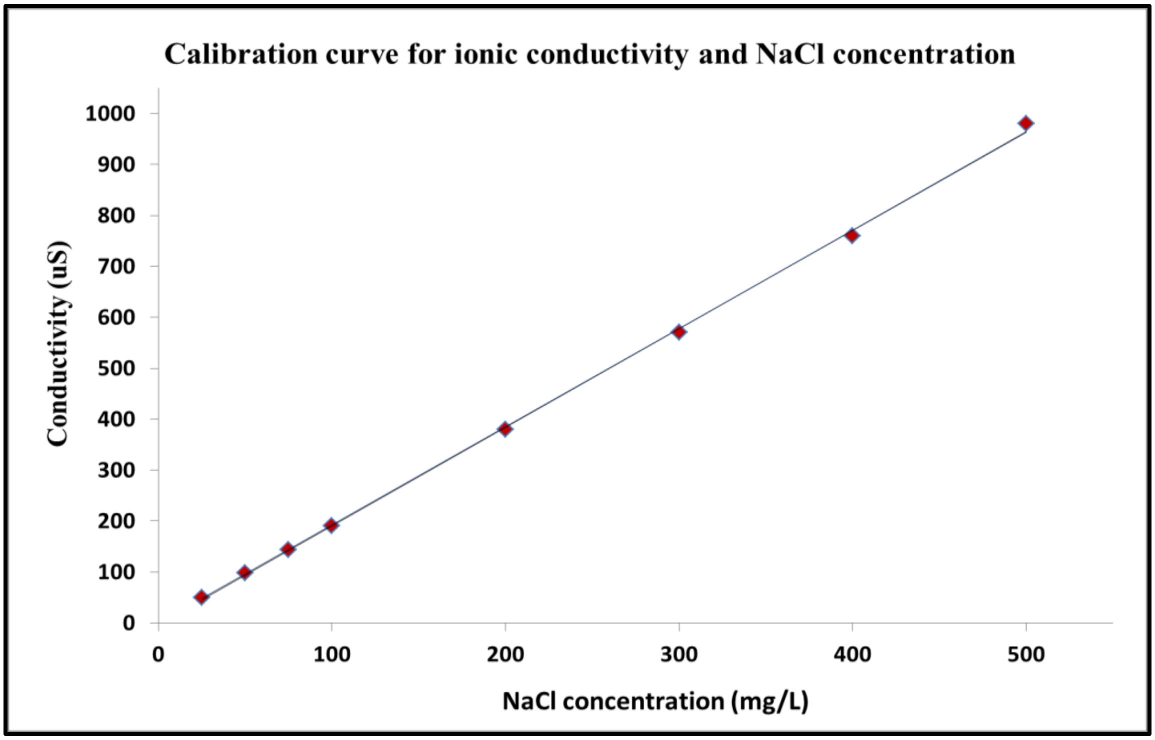

Figure S2. Calibration curve linearity for ionic conductivity vs. NaCl concentration.

Calculation of Ion Removal from NaCl Solution

The conductivity of the solution is measured by the conductivity meter. If the initial NaCl solution conductivity is 143.00 µS/cm, and after charging the electrode it became 138.59 µS/cm, then the ion removal from the solution can be calculated. As we know from Equation (S1):

$$\text{Conductivity} = 1.9067 \times \text{Concentration}$$

hence:

$$\text{Concentration} = \text{Conductivity}/1.9067$$

Therefore: the initial concentration = (143.00)/1.9067= 75.00 mg/L and

The final concentration = (138.59)/1.9067= 72.69 mg/L.

Hence, the ion removal from the NaCl solution = initial concentration − final concentration = 2.31 mg/L.

S3. Construction of a CDI Cell and Flow-Through Open Cell Fabrication

Capacitive deionization experiments were carried out in a flow-through system, depicted in Figure S3a. The CDI unit cell consisted of a flow-through cell which has two parallel electrodes that allows aqueous solution to stream between them, and the spacing of 5 mm between the electrodes is maintained.

Lawrence Livermore National Laboratories in the USA designed a CDI unit as a closed system to force water flow into all the pores of the electrode. It had a retaining plate, rubber

gasket, electrode, rubber spacer, and a nylon spacer and it was usually modified by researchers to make it suitable for their applications.

A flow-through electrode was prepared for use in this CDI cell and this electrode has low hydraulic resistance. This means that water flow will easily pass through all the pores of the electrodes in a CDI flow-through cell, as shown in Figure S3b. This cell was designed in our laboratory and it was built by printing on a Connex 350 3D printer, by Objet. This printer built our cell with UEROBACK material over a period of three hours. After that, it was washed with water in readiness for use in the CDI system. The cell production was basic, easy, and fast, very accurate, and resulted in a strong product. In this cell, we avoided using rubber gaskets, a nut tool, threaded rods, and an insulator layer between electrodes. Additionally, the electrical contact with electrodes was good and very easy. Furthermore, this cell has a rectangular external (Figure S3b) and H-like internal (Figure S3c) shape. The dimensions of the outside cell was 57 mm × 62 mm × 32 mm in height, length, and width, respectively. Each side had one 4 mm diameter hole for solution flow, one near the top and the other near the bottom. These holes are connected with a 250 mm (MasterFlex L/S® 25) pump tubing to a peristaltic pump in a recirculating fashion (Figure S3a). It is worth noting that the middle of both sides of the cell was fitted with 35 mm × 35 mm glass windows to allow light to be directed through the cell to the electrodes. This was designed to be suitable for any future studies that need to use light for excitation of the electrodes. This glass was of dielectric material, transparent, and allows all wavelengths to pass through it. Moreover, the H-shape was designed inside the cell to be suitable for holding a glass conductivity electrode (4 mm thickness) inside the cell if needed for any relevant applications. The dimensions inside the cell were 50 mm × 50 mm × 20 mm in height, length, and width, respectively, as shown in Figure S3c (the top of the cell).

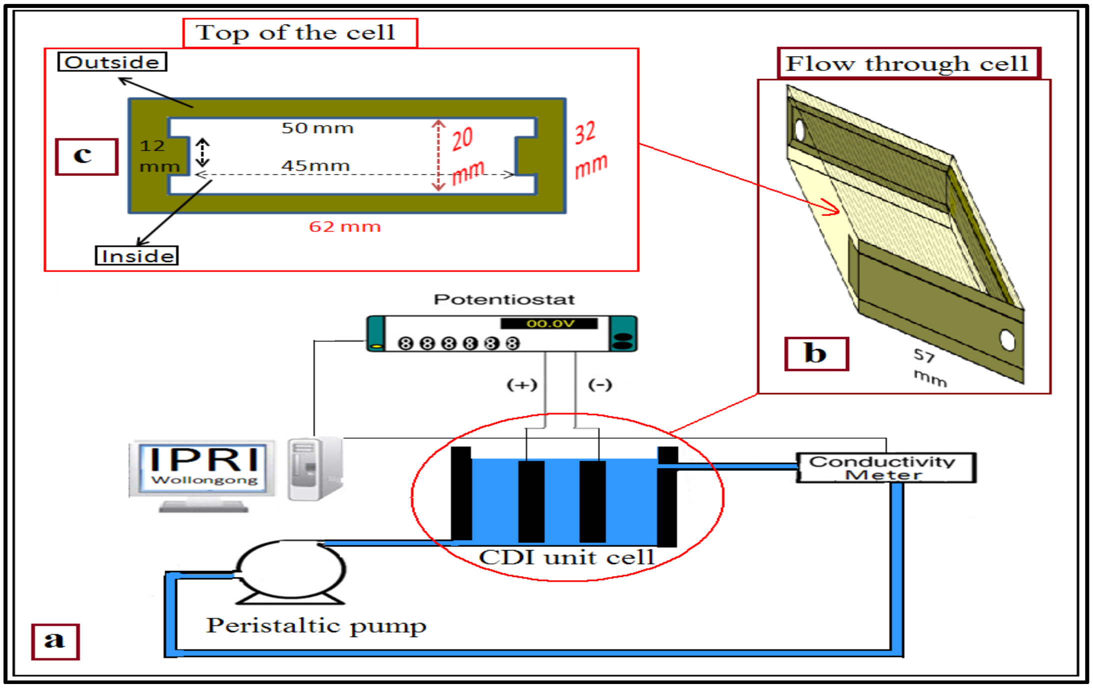

Figure S3. Schematic diagram of a capacitive deionization (CDI) cell.

S4. Calculation of Electrode Electrosorption Capacity in Terms of (mg/g) and (mg/cm^3)

The electrosorption capacity of a PEDOT coating on RVC electrodes was calculated according to the following equations [1–3]:

$$M_{mass} = [(C_0 - C_f) \times V] / m \quad (S2)$$

$$M_{volume} = [(C_0 - C_f) \times V] / Z \quad (S3)$$

$$M_{area} = [(C_0 - C_f) \times V] / A \quad (S4)$$

where M_{mass}, M_{volume}, and M_{area} are the electrosorption capacity of the working electrode in terms of mg/g, mg/cm^3, and mg/cm^2, respectively. C_0 is the initial concentration of the solution (mg/L), C_f is the final concentration of the solution (mg/L) after adsorption, V is the volume of the solution (L), m is the mass of the materials (g), and Z and A are the volume of the electrode and the geometric area, respectively.

For example, if the mass of the electrode is 0.05 g (having 2.16 cm^3 geometric volume and 17.88 cm^2 geometric area), the volume (v) and conductivity of the NaCl solution are 0.06 L and 143.00 µS/cm, respectively, and the conductivity of the NaCl solution after adsorption is 138.59 µS/cm, then the electrosorption capacities of the electrodes can be calculated. From the above section, we obtain:

Initial concentration, C_0= 75.00 mg/L and Final concentration, C_f = 72.69 mg/L.

Therefore, from Equation (S2), we obtain: $M_{mass} = [(C_0 - C_f) \times V] / m$, so, $M_{mass} = [(75 - 72.69) \times 0.06] / 0.05 = 2.77$ mg/g.

Or, from Equation (S3), we obtain: $M_{volume} = [(C_0 - C_f) \times V] /Z$, so, $M_{volume} = [(75 - 72.69) \times 0.06] /2.16 = 0.06$ mg/cm^3.

Or, from Equation (S4), we obtain: $M_{area} = [(C_0 - C_f) \times V] /Z$, so, $M_{area} = [(75 - 72.69) \times 0.06] /17.88 = 0.008$ mg/cm^2.

S5. Optimization of RVC Electrodes Coated with a-SWCNT

The general purpose of this work is the optimization of the reticulated vitreous carbon (RVC) electrodes of different porosities coated with a-SWCNT. RVC electrodes have a free void volume between 90% and 97%. Thus, RVC electrodes have a low flow resistance. Figure S4a–c show photos of three RVC electrodes with porosities of 60, 45, and 30 ppi. It is clear that the free void volume of the RVC electrodes increases with decreasing ppi grade. The average pore sizes of the RVC electrodes were calculated from SEM images by measuring the distance between the green lines in Figure S4d–f, and they were 350, 700, and 900 µm for 60, 45, and 30 ppi, respectively. RVC electrode properties are dependent on the ppi grade. If the amount of pores per inch (ppi) increases, the electrode area per unit electrode volume will increase as well. According to the previous reported properties of RVC [4,5], which include good surface area, conductivity, and good mechanical strength, it is envisaged that the RVC electrode that has the largest number of pores per inch would be the best to be used as an efficient electrode. Therefore, in order to confirm that the electrode with smaller pores is the best electrode for loading of a-SWCNT, we

investigated the influence of all electrode capacitances of RVC electrodes with different pore sizes coated with the same amount of a-SWCNT. All RVC electrodes had the same geometric volume (dimensions of 4.0 cm × 1.8 cm × 0.3 cm), and the same amount of a-SWCNT was coated, around 6 mg. The effect of different pores per inch was investigated in aqueous solution. Figure S4g shows the capacitances of all electrodes that were calculated from cyclic voltammograms at the potential scan rate of 5 mV/s using Equation (S5) [6]:

$$C_{mass} = Q \,/\, (2m\Delta V) \tag{S5}$$

where C_{mass} is the capacitance of electrode (F/g), Q is the charge (C), m is the mass (g), and V is the voltage (V). It can be seen that the highest specific capacitance was 267.24 F/g for the a-SWCNT-coated RVC electrode with 60 pores per inch, and the specific capacitance decreased with the decrease in the number of pores per inch. This is because the RVC electrode with 60 ppi has a higher surface area per volume of electrode. The RVC 60 ppi electrode coated with a-SWCNT (6 mg) will be discussed in detail in its electrochemical behavior evaluation using cyclic voltammetry. In conclusion, therefore, 60 ppi RVC electrodes were selected as substrates to load a-SWCNT for use as the electrode material.

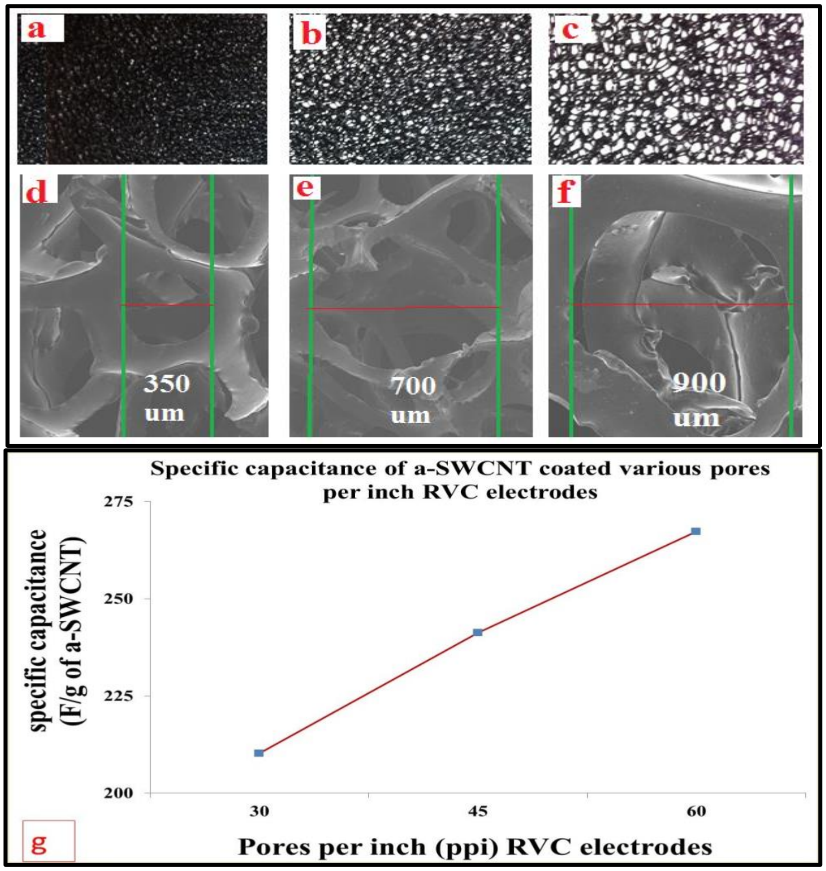

Figure S4. (**a**–**c**) Photos and (**d**–**f**) SEM micrographs of 60, 45, and 30 ppi RVC samples, respectively; (**g**) the specific capacitance of a-SWCNT-coated RVC electrodes of various porosities in 1 M NaCl solution calculated from cyclic voltammograms recorded in a voltage range between −0.2 and 1.0 V using a three-electrode system vs. Ag/AgCl at a 5-mV/s scan rate.

S6. Scanning Electron Microscopy of a-SWCNT-Coated RVC Electrodes

The surface morphology of the a-SWCNT-coated RVC electrode was examined using scanning electron microscopy (SEM). SEM images of the 23.58 wt% a-SWCNT-coated RVC electrode are shown in Figure S5. It can be concluded that the a-SWCNT dispersed very well in DMF because no aggregation is seen on the surface and RVC pores appeared to be completely filled (Figure S5a). The appearance of the top surface is like a textile (Figure S5b) because the nanotubes are irregularly spread on the RVC electrode and its pores are partly parallel and partly perpendicular to the surface. The SEM images in Figure S5c–e show that the a-SWCNT tubes became stronger and tougher due to the closer contact, which improved ion transfer between nanotubes, which is consistent with the previously-reported results [7,8]. In addition, it shows that the void spaces, or "pores", between the matted SWCNT tubes are macroscale, and these void spaces can be considered as macropores and are areas through which ion diffusion can freely take place. From the SEM images, the SWCNT nano-network structures act as useful nano-spacers for diminishing the aggregation of SWCNT. This 3D porous structure exposes an extensive surface area that facilitates ion diffusion, leading to a high performance of electrosorption. It maximizes the surface area, potentially allowing large capacitances to be obtained [9]. This will also lead to an increase in the ions' capture and the conductive properties of a-SWCNT.

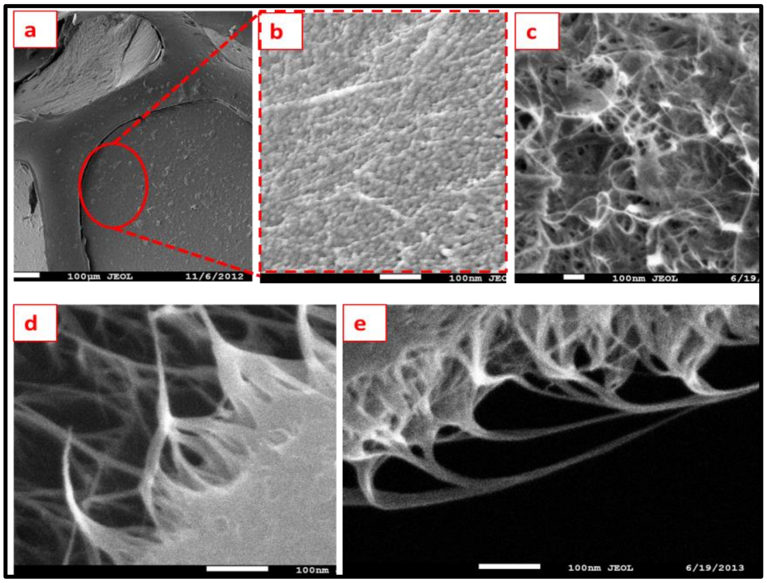

Figure S5. Scanning electron microscopy (SEM) images (**a**,**b**) top surface, (**c**) cross-section, and (**d**,**e**) 45° view of 23.58 wt% a-SWCNT loading.

S7. Electrochemical Behavior Evaluation using Cyclic Voltammetry

In order to evaluate the electrochemical properties of the a-SWCNT-coated RVC electrode, cyclic voltammetry was applied as it is one of the most widely-used techniques to study electrochemical reactions. In this section, it is used to determine the effect of filling the pores of RVC by a-SWCNT on the capacitance, the effect of increasing scan rate on electron transfer and capacitance, and the stability of the electrode using 1 M NaCl solution recorded in the voltage range between −0.2 and 1.0 V vs. Ag/AgCl in a three-electrode system with an RVC counter electrode.

S8. The Capacitance of the RVC Electrode before and after Loading with a-SWCNT

The capacitance of the RVC electrode in 1 M NaCl solution using a three-electrode system was discussed in our previous publication [10], and it was calculated to be 0.002 F/cm³ using Equation (S6) [6]:

$$C_{volume} = Q/(2Z\Delta V) \quad \text{(S6)}$$

where C_{volume} is the capacitance of the electrode (F/cm³), Q is the charge (C), Z is the geometric volume (cm³), and V is the voltage (V). Figure S6a shows the cyclic voltammograms of the 1 cm³ RVC electrode and the same electrode filled with 23.58 wt% a-SWCNT under the same conditions. The peak current (i_p) of the 23.58 wt% a-SWCNT-coated RVC electrode compared to a bare RVC electrode has increased by a factor of 1375. This is related to the large surface area of a-SWCNT compared to a bare RVC electrode, according to the Randle–Sevcik relationship [11]. The specific capacitance of the 23.58 wt% a-SWCNT-coated RVC electrode was 2.75 F/cm³. Figure S6b shows that the specific capacitance of the electrode increased with increasing amounts of a-SWCNT in geometric volume. The specific capacitance was 0.56 F/cm³, 1.02 F/cm³, and 1.31 F/cm³ for the electrode that had 3.63, 12.50, and 17.43 wt% a-SWCNT coated on the RVC electrodes, respectively. The specific capacitances of 3.63, 12.50, and 17.43 wt% a-SWCNT coated on the RVC electrodes compared to a bare RVC electrode have increased by a factor of 280, 510, and 655, respectively. Figure S6a shows the pseudo-capacitive behavior at 0.5 V for the RVC electrode coated by a-SWCNT. This behavior referred to the functional group, which was created by the nitric acid treatment on the SWCNT surface [12].

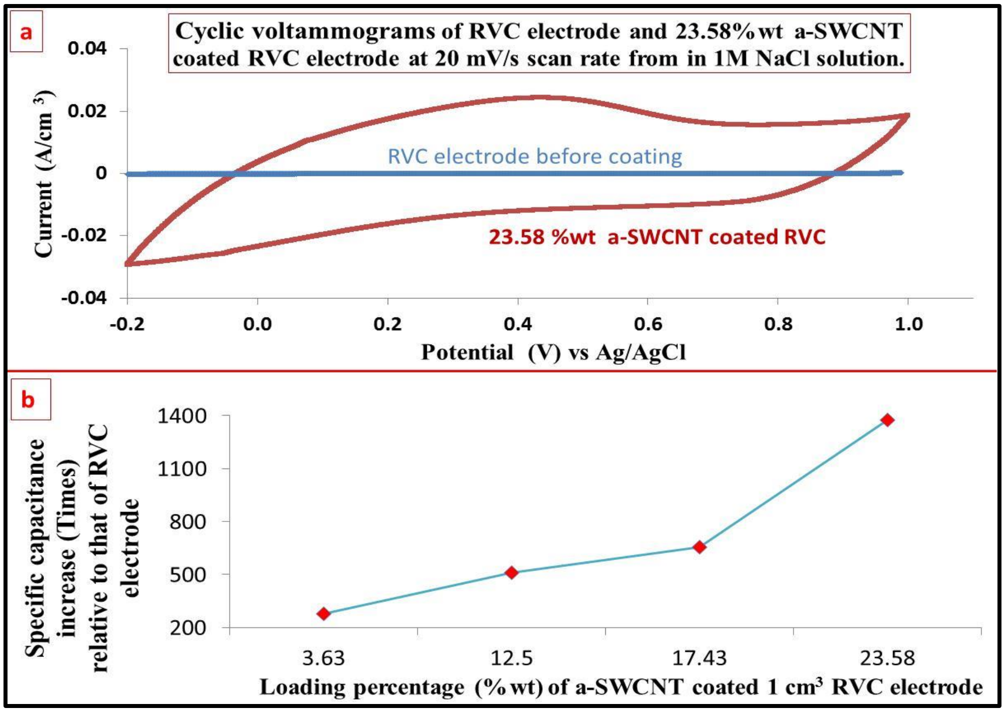

Figure S6. (**a**) Comparison cyclic voltammograms of the 1 cm³ bare RVC electrode and the same size of the 23.58 wt% a-SWCNT-coated RVC electrode in 1 M NaCl scanned at 20 mV/s and using an RVC counter electrode and Ag/AgCl reference electrode in a three-electrode system; and (**b**) the effect of a-SWCNT loading of a-SWCNT/RVC composite electrodes on specific capacitance.

S9. Effect of Increasing the Scan Rate on the Electrode Capacitance

The capacitive behavior of the a-SWCNT resulted mainly from electrochemical double-layer charging along with a negligible contribution of pseudo-capacitance. In this study, the effect of different scan rates on the electrode capacitance was investigated in 1 M NaCl aqueous solution. Figure S7a shows the cyclic voltammograms of the 3.63 wt% a-SWCNT-coated RVC working electrode obtained with various potential scan rates. It can be noted that electrochemical reactions occur in the potential range of (−0.2–0.4 V) for scan rates of 5 mV/s, resulting in redox peaks at about 0 and 0.2 V. However, the shapes of the CVs at scan rates of 5–20 mV/s are close to rectangular, and as we know, the achievement of a rectangular-shaped CV is the suggested ultimate goal in electrochemical double-layer capacitors (EDLC) [6]. The absence of Faradaic reactions at 0.5 V indicates that the current response here absolutely comes from the electric double-layer EDL formation [13]. The enhanced capacitance compared with raw SWCNT relates to the increased surface charge density from oxygen atoms with more negative electronic affinity [12]. According to the characteristics of the CV, it can be deduced that the contribution of carboxyl and carbonyl groups to CNTs is in the pseudo-capacitance, which increases the apparent capacitance [14,15]. It is clear that the polarization caused by a high scan rate leads to the anodic peaks shifting toward high potential and the cathodic peaks moving toward negative potential simultaneously [14]. However, when the scan rate was increased to 50 mV/s, the curves were characterized by a non-rectangular shape that indicated resistance-like electrochemical behavior. This leads to a continuous decrease in the capacitance of electrodes with increasing scan rate.

Figure S7b presents the capacitances of various a-SWCNT-coated RVC electrodes as a function of the scan rate. It is observed that increasing the amount of a-SWNT in the RVC electrode led to a decrease in the specific capacitance of a-SWCNT. For example, the highest specific capacitance was 267.24 F/g for 3.63 wt% a-SWCNT, and the lowest specific capacitance was 139.65 F/g for 23.58 wt% a-SWCNT at a scan rate of 5 mV/s. It seems also that a-SWCNT-coated RVC electrodes gave, in all electrodes, a high capacitance at a low scan rate, but its capacitance markedly decreased at high scan rates. For instance, the specific capacitance of 3.63 wt% a-SWCNT coated on RVC was 267.24, 239.58, 207.40, 132.69, 84.77, and 51.15 F/g at 5, 10, 20, 50, 100, and 200 mV/s, respectively (Table S1). It is expected that the capacitive volume should increase with increasing scan rates because it is found in all cases of carbon nanotubes [6,12–14,16]. This characteristic has been attributed to the resistance of the electrolyte and the inner resistance of ion diffusion with certain carbon micro-pores' surfaces being partially accessible to electrolytes. This becomes significant under relatively high scan rates due to the differential depletion of the electrolyte concentration [16–18]. It can be seen that the specific capacitance trend of the 3.63 wt% electrode sharply decreases when the scan rate is above 20 mV/s. For other electrodes, the specific capacitance decreasing trend is more pronounced when the scan rate is increased above 50 mV/s. The specific capacitance values of all electrodes were calculated using Equation (S5).

Figure S7c,d shows the cyclic voltammograms of a-SWCNT composite electrodes with different loadings of a-SWCNT in terms of current per gram of a-SWCNT and current per geometric volume of electrode, respectively. It is observed that increasing the amount of a-SWNT in the RVC electrode led to a decrease in the current per gram of a-SWCNT (Figure S7c) but, in contrast, led to an increase in the current per geometric volume of the electrode (Figure S7d). Hence, the effect of the geometric volume of the electrode on the results will now be discussed. The calculated capacitance per unit geometric volume of the porous electrode is presented in Figure S7e and Table S1 as F/cm^3. This is not the same as the per unit volume of the active

material (i.e., SWCNT) alone. The capacitance of the electrode per geometric volume (F/cm^3) was calculated using Equation (S6). Figure S7e shows that the capacitances of various a-SWCNT-coated RVC electrodes increase with the increase in the amount of a-SWCNT in the geometric volume. The specific capacitance, obtained at 20 mV/s, was 0.56, 1.02, 1.31, and 2.75 F/cm^3 for the electrode that had 3.63, 12.50, 17.43, and 23.58 wt% a-SWCNT coated on the RVC electrodes, respectively. This indicates that the surface area of a-SWCNT coated on the RVC electrode had increased. In addition, the effect of the geometric area of the electrode on the results will now be considered. The calculated capacitance per unit of geometric area of the porous electrode is presented in Table S1 as F/cm^2. The capacitance of the electrode per geometric area (F/cm^2) was calculated using Equation (S7), which can be given as [6]:

$$C_{area} = Q/(2A\Delta V) \quad \text{(S7)}$$

where C_{area} is the capacitance of the electrode (F/cm^2), Q is the charge (C), A is the geometric area (cm^2), and V is the voltage (V). The increase of the SWCNT loading level could increase the active area resulting in higher specific capacitance. In Table 3, the capacitance obtained at 20 mV/s was 0.07, 0.12, 0.16, and 0.33 F/cm^2 for the electrode that had 3.63, 12.50, 17.43, and 23.58 wt% a-SWCNT coated on the RVC electrodes, respectively. This indicates that the surface area of a-SWCNT coated on the RVC electrode had increased.

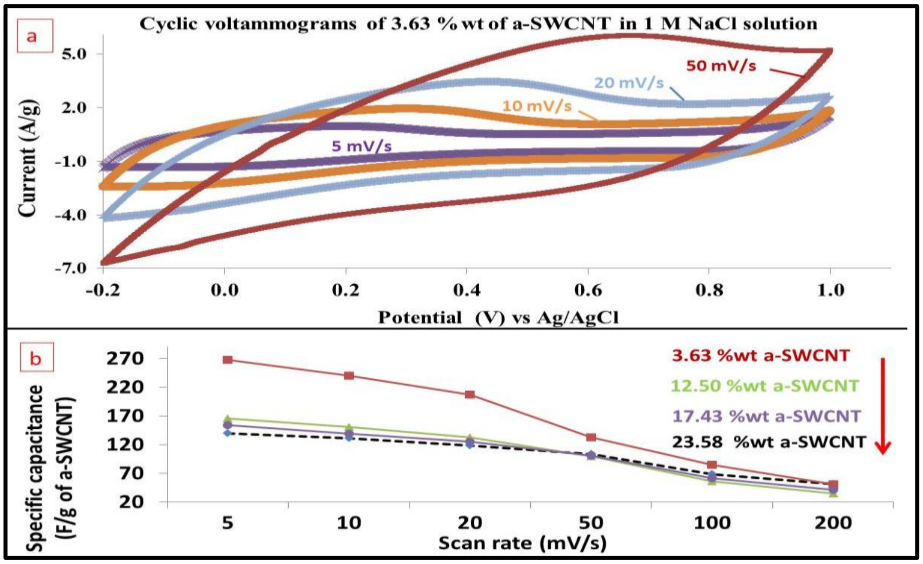

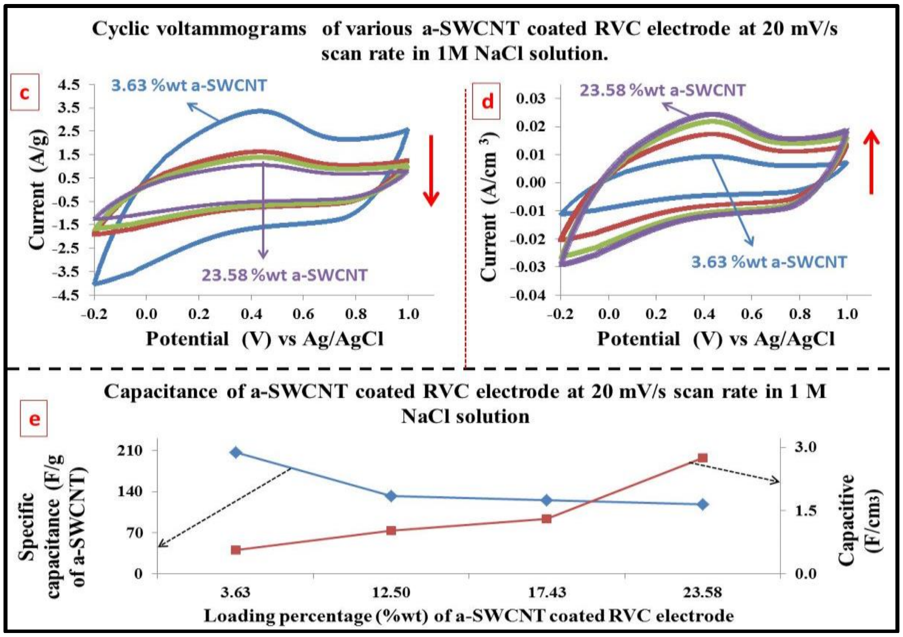

Figure S7. (**a**) Cyclic voltammograms of the 3.63 wt% a-SWCNT-coated RVC electrode and (**b**) specific capacitance of various a-SWCNT-coated RVC electrodes at various scan rates in 1 M NaCl solution in the voltage range between −0.2 and 1.0 V vs. Ag/AgCl using a three-electrode system. Cyclic voltammograms of a-SWCNT-coated RVC electrodes at 20 mV/s, in terms of (**c**) current per gram of a-SWCNT and (**d**) current per geometric volume of the electrode; and (**e**) capacitance of the electrodes per gram of a-SWCNT and per geometric volume of the electrode.

Table S1. Specific capacitance of various a-SWCNT-coated RVC electrodes by F/g of a-SWCNT, F/area of the electrode, and F/volume of the electrode, in 1 M NaCl solution at various scan rates.

**Sample**		**3.63 wt% a-SWCNT-coated RVC**					
Scan rate (mV/s)		5	10	20	50	100	200
Specific Capacitance	(F/g)	267.24	239.58	207.40	132.69	84.78	51.15
	(F/cm^2)	0.09	0.08	0.07	0.03	0.03	0.02
	(F/cm^3)	0.72	0.64	0.56	0.36	0.23	0.14
Sample		12.50 wt% a-SWCNT-coated RVC					
Scan rate (mV/s)		5	10	20	50	100	200
Specific Capacitance	(F/g)	165.20	150.33	132.16	99.12	56.17	34.69
	(F/cm^2)	0.21	0.18	0.12	0.07	0.04	0.02
	(F/cm^3)	1.76	1.46	1.02	0.55	0.31	0.17
Sample		17.43 wt% a-SWCNT-coated RVC					
Scan rate (mV/s)		5	10	20	50	100	200
Specific Capacitance	(F/g)	153.79	138.94	125.20	100.77	61.07	41.23
	(F/cm^2)	0.29	0.23	0.16	0.08	0.04	0.02
	(F/cm^3)	2.42	1.93	1.31	0.67	0.37	0.19
Sample		23.58 wt% a-SWCNT-coated RVC					
Scan rate (mV/s)		5	10	20	50	100	200
Specific Capacitance	(F/g)	139.65	131.27	117.70	103.34	68.43	51.67
	(F/cm^2)	0.39	0.37	0.33	0.29	0.19	0.14
	(F/cm^3)	3.23	3.04	2.75	2.39	1.58	1.20

S10. Calculation Enhancement Related to the Electrosorption Capacity in a New Cell Using Flow Feed Through (FT) Electrodes Compared with Flow Between (FB) the Electrodes

For the 3.63% a-SWCNT/RVC electrode, the electrosorption capacity for one desalination cycle is considered.

Thus:

Electrosorption capacity for the FT cell = 10.40 mg/g
Electrosorption capacity for the FB cell = 8.39 mg/g

Therefore:

$$\text{Enhancement} = \left(\frac{(\text{electrosorption using FT cell} - \text{electrosorption using FB cell})}{\text{electrosorption using FB cell}}\right) \times 100 \quad \text{(S8)}$$

$$= \left(\frac{(10.40 - 8.39)}{8.39}\right) \times 100 = 23.96\%.$$

S11. Weber and Morris Model for Intra-Particle Diffusion

The rate parameters for intra-particle diffusion (k_{id}) at different initial concentrations are determined using the following equation:

$$q_t = K_{id}\ (t^{0.5}) \quad \text{(S9)}$$

where K_{id} is the intraparticle diffusion rate constant, (mg/gmin$^{0.5}$). The plot q_t versus $t^{0.5}$should give a straight line.

S12. Pseudo-First-Order Equation

The equation for pseudo-first-order is:

$$\log\,(q_t - q_e) = \log\,(q_e) - \frac{K_1 t}{2.303} \quad \text{(S10)}$$

where q_t and q_e are the amounts of NaCl adsorbed (mg/g) at time t and at equilibrium, respectively, and K_1 is the rate constant of the pseudo-first-order adsorption process (min^{-1}).

S12. Pseudo-Second-Order Equation

The equation for pseudo-second-order can be written as:

$$\frac{t}{(q_t)} = \frac{1}{(h)} + \left(\frac{1}{(q_e)}\right) t \quad \text{(S11)}$$

where $h = K_2 q_e{}^2$(mg g^{-1}min^{-1}) can be regarded as the initial adsorption rate as $t \rightarrow 0$ and K_2 is the rate constant of pseudo-second-order adsorption (g mg^{-1}min^{-1}). The plot of $\frac{t}{(q_t)}$ vs. t should give a straight line if pseudo-second-order kinetics are applicable and the q_e, K_2, and h values can be determined from the slope and intercept of the plot.

S14. Langmuir and Freundlich Isotherm

Langmuir (Equation (S12)) and Freundlich isotherms (Equation (S13)) are used to fit the experimental data for electrosorption of Na^{+} and Cl^{-1} onto the electrode, respectively:

$$q = \frac{q_m K_L C}{1 + K_L C} \quad \text{(S12)}$$

$$q = K_F C^{1/n} \quad \text{(S13)}$$

where C is the equilibrium concentration (mg/L), q is the amount of adsorbed NaCl in milligrams per gram of a-SWCNT, q_m is the maximum adsorption capacity corresponding to complete monolayer coverage (mg/g). K_L is the Langmuir constant related to binding energy, K_F is the Freundlich constant related to the adsorption capacity of adsorbent, and 1/n is the indication of the tendency of the adsorbate to be adsorbed. The value of 1/n is determined from the slope of the plot. It allows understanding of the adsorption process. Generally, $1/n < 1$. A variation in the slope between 0 and 1 is associated with a chemisorption process, which is more heterogeneous as the value gets closer to 0. At high pressure $1/n = 0$, hence, the extent of adsorption becomes independent of pressure. At $1/n = 1$, a linear plot is observed. When a slope (1/n) above 1 is observed, it is consistent with cooperative adsorption.

References

1. Boparai, H.K.; Joseph, M.; O'Carroll, D.M. Kinetics and thermodynamics of cadmium ion removal by adsorption onto nano zerovalent iron particles. *J. Hazard. Mater.* **2011**, *186(1)*, 458–465.
2. Senthil Kumar, P.; Gayathri, R. Adsorption of Pb2+ ions from aqueous solutions onto Bael tree leaf powder: isotherms, kinetics and thermodynamics. *J. Eng. Sci. Technol.* **2009**, *4(4)*, 381–399.
3. Purdom, P.W. Environmental Health. second ed. **1980**, New York: Academic Press.
4. Friedrich, J.M. Reticulated vitreous carbon as an electrode material. *J. Electroanal. Chem. Interfacial Electrochem.* **2004**, *561*, 203–217.
5. Tondi, G.; Fierro, V.; Pizzi, A.; Celzard, A. Tannin-based carbon foams. *Carbon* **2009**, *47*, 1480–1492.
6. Chen, J.H.; Li, W.Z.; Wang, D.Z.; Yang, S.X.; Wen, J.G.; Ren, Z.F. Electrochemical characterization of carbon nanotubes as electrode in electrochemical double-layer capacitors. *Carbon* **2002**, *40*, 1193–1197.
7. Zhang, X.; Li, Q.; Tu, Y.; Li, Y.; Coulter, J.Y.; Zheng, L.; Zhao, Y.; Jia, Q.; Peterson, D.E.; Zhu, Y. Strong Carbon-Nanotube Fibers Spun from Long Carbon-Nanotube Arrays. *Small* **2007**, *3*, 244–248.
8. Zhang, X.; Li, Q.; Holesinger, T.G.; Arendt, P.N.; Huang, J.; Kirven, P.D.; Clapp, T.G.; DePaula, R.F.; Liao, X.; Zhao, Y.; et al. Ultrastrong, Stiff, and Lightweight Carbon-Nanotube Fibers. *Adv. Mater.* **2007**, *19*, 4198–4201.
9. Naoi, K.; Simon, P. New Materials and New Configurations for Advanced Electrochemical Capacitors. *J. Electrochem. Soc.* **2008**, *17*, 34–37.
10. Aldalbahi, A.; Rahaman, M.; Almoiqli, M. A Strategy to Enhance the Electrode Performance of Novel Three-Dimensional PEDOT/RVC Composites by Electrochemical Deposition Method. *Polymers* **2017**, *9*, 157.
11. Bard, A. *Electrochemical Methods: Fundamentals and Applications*, 2nd ed.; Bard, A.J., Faulkner, L.R., Eds.; John Wiley: New York, NY, USA, 2001.
12. Nian, Y.R.; Teng, H.S. Nitric acid modification of activated carbon electrodes for improvement of electrochemical capacitance. *J. Electrochem. Soc.* **2002**, *149*, A1008–A1014.
13. Wang, D.; Li, F.; Liu, M.; Cheng, H. Improved capacitance of SBA-15 templated mesoporous carbons after modification with nitric acid oxidation. *New Carbon Mater.* **2007**, *22*, 307–314.

14. Li, L.; Li, F.; Xiao, Y.; Aigin, Z. The effect of carbonyl, carboxyl and hydroxyl groups on the capacitance of carbon nanotubes. *New Carbon Mater.* **2012**, *26*, 224–228.
15. Oda, H.; Yamashita, A.; Minoura, S.; Okamoto, M.; Morimoto, T. Modification of the oxygen-containing functional group on activated carbon fiber in electrodes of an electric double-layer capacitor. *J. Power Sources* **2006**, *158*, 1510–1516.
16. Teng, H.; Chang, Y.J.; Hsieh, C.T. Performance of electric double-layer capacitors using carbons prepared from phenol-formaldehyde resins by KOH etching. *Carbon* **2001**, *39*, 1981–1987.
17. Mitali, S.; Soma, D.; Monica, D. A Study of Effect of Electrolytes on the Capacitive Properties of Mustard Soot Containing Multiwalled Carbon Nanotubes. *Res. J. Chem. Sci.* **2011**, *1*, 109–113.
18. Guo, Y.; Qi, J.; Jiang, Y.; Yang, S.; Wang, Z.; Xu, H. Performance of electrical double layer capacitors with porous carbons derived from rice husk. *Mater. Chem. Phys.* **2003**, *80*, 704–709.
